# Glucagon‐like‐peptide‐1 receptor agonists versus dipeptidyl peptidase‐4 inhibitors and cardiovascular outcomes in diabetes in relation to achieved glycemic control. A Danish nationwide study

**DOI:** 10.1111/1753-0407.13560

**Published:** 2024-05-16

**Authors:** Bochra Zareini, Katrine Kold Sørensen, Ulrik Pedersen‐Bjergaard, Emil Loldrup Fosbøl, Lars Køber, Christian Torp‐Pedersen

**Affiliations:** ^1^ Department of Cardiology North Zealand University Hospital Hillerød Denmark; ^2^ Department of Endocrinology and Nephrology North Zealand Hospital Hillerød Denmark; ^3^ Department of Clinical Medicine University of Copenhagen Copenhagen Denmark; ^4^ Department of Cardiology, Rigshospitalet University of Copenhagen Copenhagen Denmark; ^5^ Department of Public Health University of Copenhagen Copenhagen Denmark

**Keywords:** DPP‐4i, GLP‐1 RA, glycemic control, HbA1c, MACE, myocardial infarction, stroke

## Abstract

**Aim:**

To compare the cardiovascular preventive effect associated with glucagon‐like‐peptide‐1 receptor agonists (GLP‐1 RA) versus dipeptidyl peptidase‐4 inhibitors (DPP‐4i) according to the achieved target level of glycated hemoglobin (HbA1c).

**Methods:**

We used retrospective Danish registries to include type 2 diabetes patients already in metformin treatment initiating GLP‐1 RA or DPP‐4i between 2007 and 2021. Patients were included 6 months after GLP‐1 RA or DPP‐4i initiation. The last available HbA1c measurement before inclusion was collected. The achieved HbA1c level was categorized according to a target level below or above 53 mmol/mol (7%). The primary outcome was a composite of nonfatal myocardial infarction, nonfatal stroke, and all‐cause death. We used a multivariable Cox proportional hazard model to estimate the effect of HbA1c levels on the outcome among GLP‐1 RA users compared to DPP‐4i users.

**Results:**

The study included 13 634 GLP‐1 RA users (median age 56.9, interquartile range [IQR]: 48.5–65.5; 53% males) and 39 839 DPP‐4i users (median age 63.4, IQR: 54.6–71.8; 61% males). The number of GLP‐1 RA and DPP‐4i users according to achieved HbA1c levels were as follows: HbA1c ≤ 53 mmol/mol (≤7.0%): 3026 (22%) versus 4824 (12%); HbA1c > 53 mmol/mol (>7.0%): 6577 (48%) versus 17 508 (44%); missing HbA1c: 4031 (30%) versus 17 507 (44%). During a median follow‐up of 5 years (IQR: 2.6–5.0), 954 GLP‐1 RA users experienced the primary outcome compared to 7093 DPP‐4i users. The 5‐year risk (95% confidence interval [CI]) of the outcome associated with GLP1‐RA versus DPP‐4i according to HbA1c categories was as follows: HbA1c ≤ 53 mmol/mol: 10.3% (8.2–12.3) versus 24.3% (22.7–25.8); HbA1c > 53 mmol/mol: 16.0% (14.3–17.6) versus 21.1% (20.3–21.9); missing HbA1c: 17.1% (15.7–18.5) versus 25.6% (24.9–26.3). The preventive effect associated with GLP‐1 RA versus DPP‐4i was significantly enhanced when achieving lower HbA1c levels: HbA1c ≤ 53 mmol/mol: 0.65 (0.52–0.80); HbA1c > 53 mmol/mol: 0.92 (0.83–1.03); missing HbA1c: 0.92 (0.84–1.02) (*p* value for interaction <.001).

**Conclusion:**

GLP‐1 RA use was associated with a lower rate of major adverse cardiovascular outcomes. The association was stronger in patients achieving the target glycemic level and weaker in patients not achieving the target glycemic level, suggestive of an interaction between achieved HbA1c level and GLP‐1 RA.

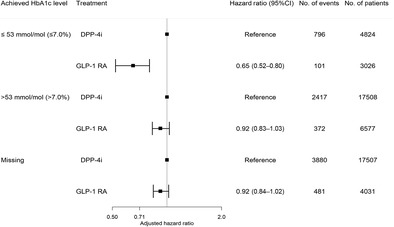

## Graphical abstract

Highlights

- Question: Does achieving the target glycemic level while on treatment with glucagon‐like‐peptide‐1 receptor agonists (GLP‐1 RA) compared to dipeptidyl peptidase‐4 inhibitors (DPP‐4i) reduce the rate of major adverse cardiovascular outcomes (MACE)?
- Findings: The rate of MACE among GLP‐1 RA versus DPP‐4i users was significantly reduced when achieving lower HbA1c levels: HbA1c ≤ 53 mmol/mol: 0.65 (0.52–0.80); HbA1c > 53 mmol/mol: 0.92 (0.83–1.03); missing HbA1c: 0.92 (0.83–1.03).
- Meaning: GLP‐1 RA use was associated with a lower rate of MACE. The association was stronger in patients achieving the target glycemic level and weaker in patients not achieving the target glycemic level, suggestive of an interaction between achieved HbA1c level and GLP‐1 RA.

## 1 INTRODUCTION

Type 2 diabetes (T2D) patients are at high risk of developing cardiovascular disease and historically medical management has focused on preventing or delaying the development of cardiovascular disease through control of glycated hemoglobin (HbA1c) levels, blood pressure, and cholesterol levels.^1^ The large cardiovascular outcome trials have, however, introduced a shift in perspective by demonstrating the preventive capabilities of glucagon‐like‐peptide‐1 receptor agonists (GLP‐1 RAs) across a range of baseline HbA1c values, prompting a reevaluation of the significance of achieving precise glycemic control.^2^ The cardiovascular outcome trials have been specifically designed to evaluate cardiovascular safety profiles of GLP‐1 RAs.^3^ To overcome the confounding effects of the HbA1c reduction, these trials were designed to promote “glycemic equipoise,” maintaining consistent glycemic levels across treatment arms.^3^ Despite efforts, glycemic equipoise was not achieved, leading to a more substantial decrease in HbA1c observed in the GLP‐1 RA arm compared to the placebo arm.^4^, ^5^, ^6^, ^7^, ^8^, ^9^, ^10^ The extent to which these differences in glycemic control directly or indirectly influenced the observed outcomes became a central question. Post‐hoc meta‐regression analyses based on trial data have shown that the effect of GLP‐1 RA on major adverse cardiovascular outcomes (MACE) was enhanced among patients with higher absolute HbA1c reduction.^11^, ^12^ To ensure statistical power, the GLP‐1 RA trials enrolled T2D patients with longer diabetes duration and higher levels of glycemic dysregulation; mean baseline HbA1c ranged from 55 mmol/mol (7.2%) to 74 mmol/mol (8.7%).^4^, ^5^, ^6^, ^7^, ^8^, ^9^, ^10^

However, the preventive effect of GLP‐1 RA of T2D patients according to achievement of the recommended HbA1c target (<53 mmol/mol, 7.0%) level has not been explored in randomized trials. Therefore, the objective of this study was to utilize the nationwide Danish registries to compare the 5‐year risk of MACE associated with GLP‐1 RA versus dipeptidyl peptidase‐4 inhibitors (DPP‐4i) treatment according to the achieved level of glycemic control.

## 2 METHODS

### 2.1 Data sources

In Denmark, an identification number given at birth or immigration allows for cross‐linkage of health and administrative databases at the individual level and enables complete follow‐up.^13^ Every citizen, including primary and hospital care, is granted equal access to the health care system. Data for this study was cross‐linked using seven nationwide registries: (a) The Danish National Patient Registry contains information on all hospital admissions and outpatient contacts from 1977 onwards. According to the *International Classification of Diseases*, *Ninth* and *Tenth Revision*, each contact is coded with a primary diagnosis and one or more secondary diagnoses. Surgical procedures are coded using the Nordic Medico‐Statistical Committee Classification of Surgical Procedures.^14^, ^15^ (b) The Danish National Prescription Registry holds information (dosage, dates, and anatomical therapeutic chemical codes) on all prescriptions dispensed from a pharmacy since 1995.^16^ (c) The Danish Cause of Death Registry entails information on the date, cause, and place of death from 1970 onwards.^17^ (d) The Danish Population Registry contains information on the date of birth and sex.^18^ (e) Information on the living situation and educational level was extracted from the Civil Registration System and the Educational register.^19^ (f) An extensive collection of laboratory databases covers most blood tests from hospitals and general practitioners in four of five regions in Denmark.^13^ (g) The Danish Adult Diabetes Registry contains information on body mass index, smoking status, and HbA1c measurements on T2D patients managed by general practitioners or the hospitals.^20^

### 2.2 Study population

We included all incident GLP‐1 RA and DPP‐4i users between 2007 and 2021 >18 years who initiated GLP‐1 RA or DPP‐4i as a second‐line T2D treatment following metformin. Treatment with DPP‐4i was selected as an active comparator because it is a frequently used second‐line therapy for T2D, exerts its effect on the blood sugar through GLP‐1, but has a neutral effect on cardiovascular outcomes.

Patients were included 6 months after treatment initiation. We excluded patients who started other glucose‐lowering drugs (including insulin), who were not in metformin treatment at the time of inclusion, who emigrated during the study period, who immigrated to Denmark in the 10 years before inclusion, who were diagnosed with end‐stage renal disease or were affected with a condition that would make HbA1c invalid as a monitoring measure (see Tables S1 and S2 in supplementary material).

### 2.3 Achieved glycated hemoglobin levels

Available HbA1c measurements within 6 months after treatment initiation were included. We used the latest measures for each patient if several measurements were available. The primary exposure of interest was the achieved HbA1c level, categorized according to two groups: ≤53 mmol/mol (≤7.0%) or >53 mmol/mol (>7.0%). Patients with a missing post‐treatment HbA1c were categorized into one group to investigate the association among those patients with a missing HbA1c value.

### 2.4 Outcome

The primary outcome was a composite outcome ofMACE: non‐fatal myocardial infarction, nonfatal stroke, and all‐cause death.

### 2.5 Follow‐up

Patients were included 6 months after treatment initiation and followed until the outcome, emigration, end of study (31 December 2021), or 5 years in total, whichever came first.

### 2.6 Comorbidities and educational level

Comorbidities were identified from before in‐ and out‐hospital contacts up to 10 years before the date of inclusion. Information on concomitant medical therapy was obtained from dispensed prescriptions listed in the Danish National Prescription Registry and defined by at least one redeemed prescription 6 months before the inclusion date. Hypertension was defined as treatment with at least two classes of antihypertensive drugs 6 months before inclusion, as done previously.^21^ Educational level was defined as the highest level of completed education and classified according to the International Standard Classification of Education. Missing information on the educational level was classified as basic level education.

### 2.7 Statistical analysis

Baseline characteristics were described using proportions for categorical variables and means and SDs or medians and interquartile ranges (IQR) for continuous variables. A reverse Kaplan–Meier estimator was used to estimate the median follow‐up time.^22^ We used the Kaplan–Meier estimator to assess the 5‐year risk of the primary outcome of MACE and the Aalen–Johansen estimator to calculate the risk of individual components of the outcome when death was present as a competing risk (ie, nonfatal myocardial infarction and nonfatal stroke). To investigate how different levels of achieved HbA1c affected the rate of the outcome among GLP1‐RA users compared to DPP‐4i users, a multivariate Cox‐proportional model was estimated with an interaction term between treatment and achieved HbA1c level. The model included the following covariates: age, gender, living alone (yes/no), T2D duration, pretreatment HbA1c category (≤53 mmol/mol [≤7.0%], >53 mmol/mol [>7.0%], or missing HbA1c), highest achieved educational level, body mass index category (underweight/normal weight, overweight, missing), smoking status (never smoker, ex‐smoker, occasional, daily smoker, missing), known comorbidities (atrial fibrillation, cancer, chronic obstructive pulmonary disease, hypertension, chronic kidney disease, cardiovascular disease, heart failure), and inclusion year. To investigate potential differences across subgroups, we reproduced these analyses in subgroups of specific patient characteristics (gender, age category, presence of cardiovascular disease at baseline (including heart failure), and inclusion year) to assess whether the interaction of achieved HbA1c category and treatment on the outcome persisted. A cause‐specific Cox regression model was used to investigate the individual components of the composite outcome where death was a competing risk. To estimate how different levels of achieved HbA1c were associated with the rate of the outcome, an interaction term between treatment and achieved HbA1c level was included. The model was adjusted for the same covariates mentioned. All estimates are reported with 95% confidence intervals (CIs).

### 2.8 Supplementary analyses

To assess the robustness of the primary analyses, we performed the following analyses:

1. The primary analysis was repeated with a finer categorization of HbA1c levels according to four groups: <48 mmol/mol (<6.5%), 48–53 mmol/mol (6.5%–6.9%), 54–58 mmol/mol (7.0%–7.5%), and <58 mmol/mol (>7.5%). Important to note that 47 and 48 mmol/mol are converted to 6.5% according to the National Glycohemoglobin Standardization Program HbA1c.
2. To ensure the generalizability of our results, we repeated the primary analyses, including cardiovascular death instead of all‐cause death in the composite outcome of MACE. In the Cause of Death registry, registration of cardiovascular death is available only up to 31 December 2018; therefore, we repeated the primary analysis, including incident GLP‐1 RA or DPP‐4i users between 2007 and 2018. Patients were included 6 months following treatment initiation and followed until the outcome, emigration, end of study (31 December 2018), or 5 years in total, whichever came first.
3. To ensure that the achieved HbA1c level was a consequence of the treatment, we restricted the study population to those with high adherence to GLP1‐RA and DPP‐4i before inclusion. This was done by calculating the treatment duration for each drug based on the available prescriptions. A patient was considered to be under treatment from the first prescription until the date of the subsequent prescription. If no additional prescription was obtained, a treatment duration of 3 months was assumed. Only persons with a proportion of days covered above 80% within the 6 months before inclusion were included.
4. To investigate how the cumulative exposure of increased blood sugar levels could affect the risk of MACE among GLP‐1 RA users compared to DPP‐4i users, we used the mean HbA1c value to represent the cumulative exposure. Available HbA1c measurements were collected within 6 months before and after treatment initiation with GLP‐1 RA or DPP‐4i. If several measurements were present, the earliest value before treatment (but within the 6 months) and the latest value following treatment (within the 6 months) were collected. Each mean HbA1c value was categorized according to the same categories mentioned earlier. Patients with a missing pretreatment and/or post‐treatment HbA1c compromised their categories.
5. GLP‐1 RA was introduced in Denmark in 2007–2012 and this could affect which patients would be treated with DPP‐4i, sulfonylurea, and thiazolidinedione following 2012. To investigate if our association was confounded by this, we created a cohort including only patients in treatment with DPP‐4i, sulfonylurea, and thiazolidinedione prior to 2012 and patients in treatment with GLP‐1 RA included after 2013. The primary analyses were repeated using DPP‐4i or sulfonylurea and thiazolidinedione as the reference group.

All analyses were performed in R version 4.2.1.^23^

### 2.9 Ethics

By law, registered‐based research has been granted permission to be performed without ethical approval or informed consent. Therefore, the project has been approved by the Knowledge Centre on Data Protection Compliance‐The Capitol Region of Denmark (approval number: P‐2010‐191).

## 3 RESULTS

### 3.1 Characteristics of the study population

**FIGURE 1:**
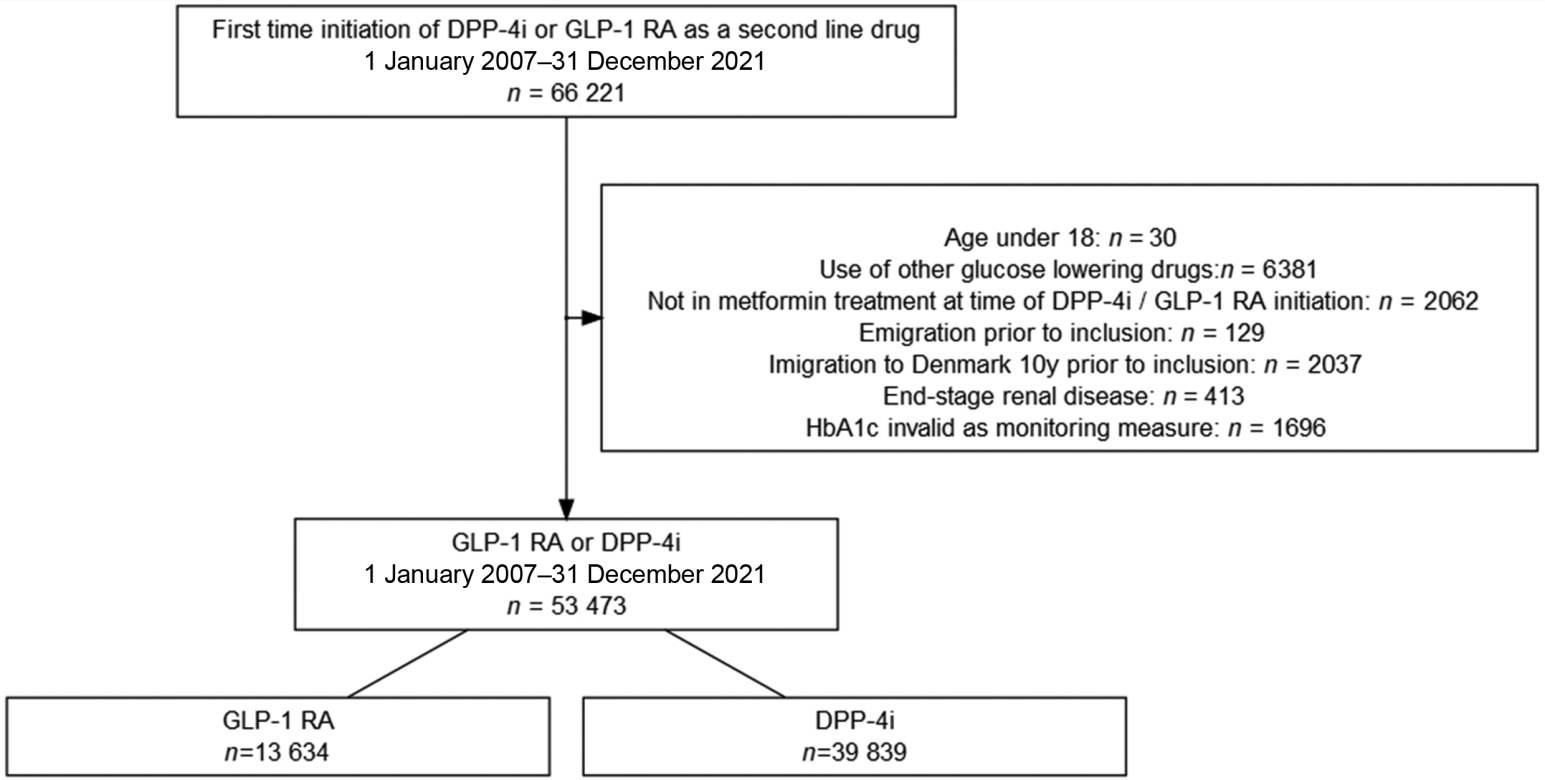
Flowchart of the study cohort. DPP‐4i, dipeptidyl peptidase‐4 inhibitor; GLP‐1 RA, glucagon‐like‐peptide‐1 receptor agonists; HbA1c, glycated hemoglobin; n, number.

The final study population consisted of 53 473 T2D patients, of whom the following HbA1c distribution among GLP‐1 RA and DPP‐4i users was achieved: HbA1c ≤ 53 mmol/mol (≤7.0%): 3026 (22%) versus 4824 (12%); HbA1c > 53 mmol/mol (>7.0%): 6577 (48%) versus 17 508 (44%); missing HbA1c: 4031 (30%) versus 17 507 (44%), as shown in Figure 1. The frequency distribution of all HbA1c values according to treatment is shown in Figure S1 in the supplementary material. Patients in treatment were GLP‐1 RA were more likely to be treated with liraglutide (50.9%) or liraglutide (46.5%) than another type of GLP‐1 RAs (2.6%). Characteristics of the study population according to achieved HbA1c level are shown in Table 1. Patients treated with GLP‐1 RA were more likely to be female, younger, not living alone, better educated, overweight, and active/ex‐smokers, to have shorter diabetes duration and better kidney function compared to DPP‐4i users across all levels of achieved HbA1c category. Second, they were less likely to be diagnosed with comorbidities and receive concomitant medication than DPP‐4i users. No differences in the percentage of baseline cardiovascular disease were observed between GLP‐1 RA and DPP‐4i users within each HbA1c category.

**TABLE 1:** Characteristics of the study population at the time of inclusion.

		HbA1c ≤ 53 mmol/mol (≤7.0%)		HbA1c > 53 mmol/mol (>7.0%)		HbA1c missing	
		DPP‐4i (*n* = 4824)	GLP‐1 RA (*n* = 3026)	DPP‐4i (*n* = 17 508)	GLP‐1 RA (*n* = 6577)	DPP‐4i (*n* = 17 507)	GLP‐1 RA (*n* = 4031)
Age (years)	Median (IQR)	67.2 (58.3–74.3)	57.4 (48.1–66.5)	63 (54.2–71.9)	57.2 (49.3–65.5)	62.8 (54.1–70.8)	56 (47.4–64.8)
Males		2547 (52.8)	1220 (40.3)	11 031 (63.0)	3898 (59.3)	10 721 (61.2)	2109 (52.3)
T2D duration (years)	Median (IQR)	3.3 (1.4–6.1)	2.6 (1.1–5.6)	3.8 (1.4–6.6)	3.2 (1.1–6.1)	2.9 (1.1–5.3)	2.9 (1.3–5.4)
Pretreatment HbA1c	≤53 mmol/mol (≤ 7.0%)	3074 (63.7)	1992 (65.8)	6509 (37.2)	3312 (50.4)	1686 (9.6)	797 (19.8)
	>53 mmol/mol (>7.0%)	378 (7.8)	56 (1.9)	6674 (38.1)	1593 (24.2)	1302 (7.4)	329 (8.2)
	Missing	1372 (28.4)	978 (32.3)	4325 (24.7)	1672 (25.4)	14 519 (82.9)	2905 (72.1)
Highest attained education	Basic education	1860 (38.6)	919 (30.4)	6882 (39.3)	2251 (34.2)	7369 (42.1)	1435 (35.6)
	General upper secondary education	2086 (43.2)	1362 (45.0)	7590 (43.4)	3067 (46.6)	7422 (42.4)	1797 (44.6)
	Bachelor level education	686 (14.2)	588 (19.4)	2401 (13.7)	1035 (15.7)	2191 (12.5)	637 (15.8)
	Master's or PhD	192 (4.0)	157 (5.2)	635 (3.6)	224 (3.4)	525 (3.0)	162 (4.0)
Living alone		736 (15.3)	221 (7.3)	2238 (12.8)	486 (7.4)	4491 (25.7)	829 (20.6)
BMI category	Underweight/normal weight	206 (4.3)	33 (1.1)	565 (3.2)	102 (1.6)	267 (1.5)	26 (0.6)
	Overweight/obese	1119 (23.2)	943 (31.2)	4358 (24.9)	2374 (36.1)	2166 (12.4)	879 (21.8)
	Missing	3499 (72.5)	2050 (67.7)	12 585 (71.9)	4101 (62.4)	15 074 (86.1)	3126 (77.5)
Smoking status	Ex smoker	1022 (21.2)	450 (14.9)	3433 (19.6)	1190 (18.1)	2984 (17.0)	742 (18.4)
	Never smoker	593 (12.3)	416 (13.7)	2130 (12.2)	957 (14.6)	1897 (10.8)	523 (13.0)
	Occasional or daily smoker	364 (7.5)	192 (6.3)	1386 (7.9)	586 (8.9)	1315 (7.5)	343 (8.5)
	Missing	2845 (59.0)	1968 (65.0)	10 559 (60.3)	3844 (58.4)	11 311 (64.6)	2423 (60.1)
eGFR ml/min/1.73 m2	30–59	544 (11.3)	133 (4.4)	1561 (8.9)	290 (4.4)	361 (2.1)	48 (1.2)
	60–89	926 (19.2)	449 (14.8)	3643 (20.8)	975 (14.8)	739 (4.2)	190 (4.7)
	>90	489 (10.1)	354 (11.7)	2620 (15.0)	1078 (16.4)	463 (2.6)	166 (4.1)
	Missing	2865 (59.4)	2090 (69.1)	9684 (55.3)	4234 (64.4)	15 944 (91.1)	3627 (90.0)
LDL mmol/L	0–2.5	1199 (24.9)	681 (22.5)	4647 (26.5)	1488 (22.6)	745 (4.3)	253 (6.3)
	>2.5	442 (9.2)	247 (8.2)	1534 (8.8)	502 (7.6)	291 (1.7)	109 (2.7)
	Missing	3183 (66.0)	2098 (69.3)	11 327 (64.7)	4587 (69.7)	16 471 (94.1)	3669 (91.0)
Hypertension		1687 (35.0)	943 (31.2)	5871 (33.5)	2182 (33.2)	6760 (38.6)	1454 (36.1)
Atrial fibrillation		364 (7.5)	168 (5.6)	1207 (6.9)	367 (5.6)	1064 (6.1)	186 (4.6)
Cancer		497 (10.3)	183 (6.0)	1498 (8.6)	401 (6.1)	1286 (7.3)	214 (5.3)
COPD/asthma		701 (14.5)	490 (16.2)	2232 (12.7)	892 (13.6)	2368 (13.5)	625 (15.5)
CKD		436 (9.0)	82 (2.7)	648 (3.7)	238 (3.6)	634 (3.6)	114 (2.8)
Cardiovascular disease		1362 (28.2)	854 (28.2)	4747 (27.1)	1816 (27.6)	4872 (27.8)	1108 (27.5)
Stroke		280 (5.8)	103 (3.4)	937 (5.4)	284 (4.3)	878 (5.0)	151 (3.7)
Heart failure		313 (6.5)	118 (3.9)	839 (4.8)	247 (3.8)	769 (4.4)	171 (4.2)
PAD		185 (3.8)	62 (2.0)	556 (3.2)	145 (2.2)	609 (3.5)	101 (2.5)
ASA		1236 (25.6)	512 (16.9)	4005 (22.9)	1197 (18.2)	5425 (31.0)	1044 (25.9)
Statins		3436 (71.2)	1846 (61.0)	12 179 (69.6)	4366 (66.4)	12 393 (70.8)	2576 (63.9)
ACE/ARB		2949 (61.1)	1716 (56.7)	10 535 (60.2)	3996 (60.8)	10 955 (62.6)	2381 (59.1)
Beta‐blockers		1393 (28.9)	641 (21.2)	4263 (24.3)	1434 (21.8)	4394 (25.1)	951 (23.6)
Calcium channel blockers		1452 (30.1)	779 (25.7)	4570 (26.1)	1777 (27.0)	4882 (27.9)	1005 (24.9)
Thiazide		692 (14.3)	459 (15.2)	2180 (12.5)	935 (14.2)	2483 (14.2)	599 (14.9)
MRA		276 (5.7)	198 (6.5)	718 (4.1)	329 (5.0)	641 (3.7)	216 (5.4)
Digoxin		171 (3.5)	27 (0.9)	639 (3.6)	151 (2.3)	610 (3.5)	66 (1.6)
Loop diuretics		784 (16.3)	378 (12.5)	1930 (11.0)	693 (10.5)	2080 (11.9)	458 (11.4)
ADPi		1482 (30.7)	628 (20.8)	4854 (27.7)	1470 (22.4)	5938 (33.9)	1131 (28.1)
Vitamin K antagonist		249 (5.2)	78 (2.6)	768 (4.4)	164 (2.5)	915 (5.2)	148 (3.7)
NOACs		230 (4.8)	137 (4.5)	709 (4.0)	304 (4.6)	248 (1.4)	75 (1.9)

Abbreviations: ACE, angiotensin‐converting enzyme; ADP, adenin diphosphate receptor; ARB, angiotensin II receptor blocker; BMI, body mass index; CKD, chronic kidney disease; COPD, chronic obstructive pulmonary disease; DPP‐4, dipeptidyl peptidase‐4; eGFR, estimated glomerular filtration rate; GLP‐1, glucagon‐like peptide‐1; HbA1c, glycated hemoglobin; IQR, interquartile range; LDL, low‐density lipoprotein; MRA, mineralocorticoid receptor antagonists; n, number; NOACs, new oral anticoagulants; PAD, peripheral artery disease; T2D, type 2 diabetes.

### 3.2 Association of HbA1c level and risk of primary cardiovascular outcomes

**FIGURE 2:**
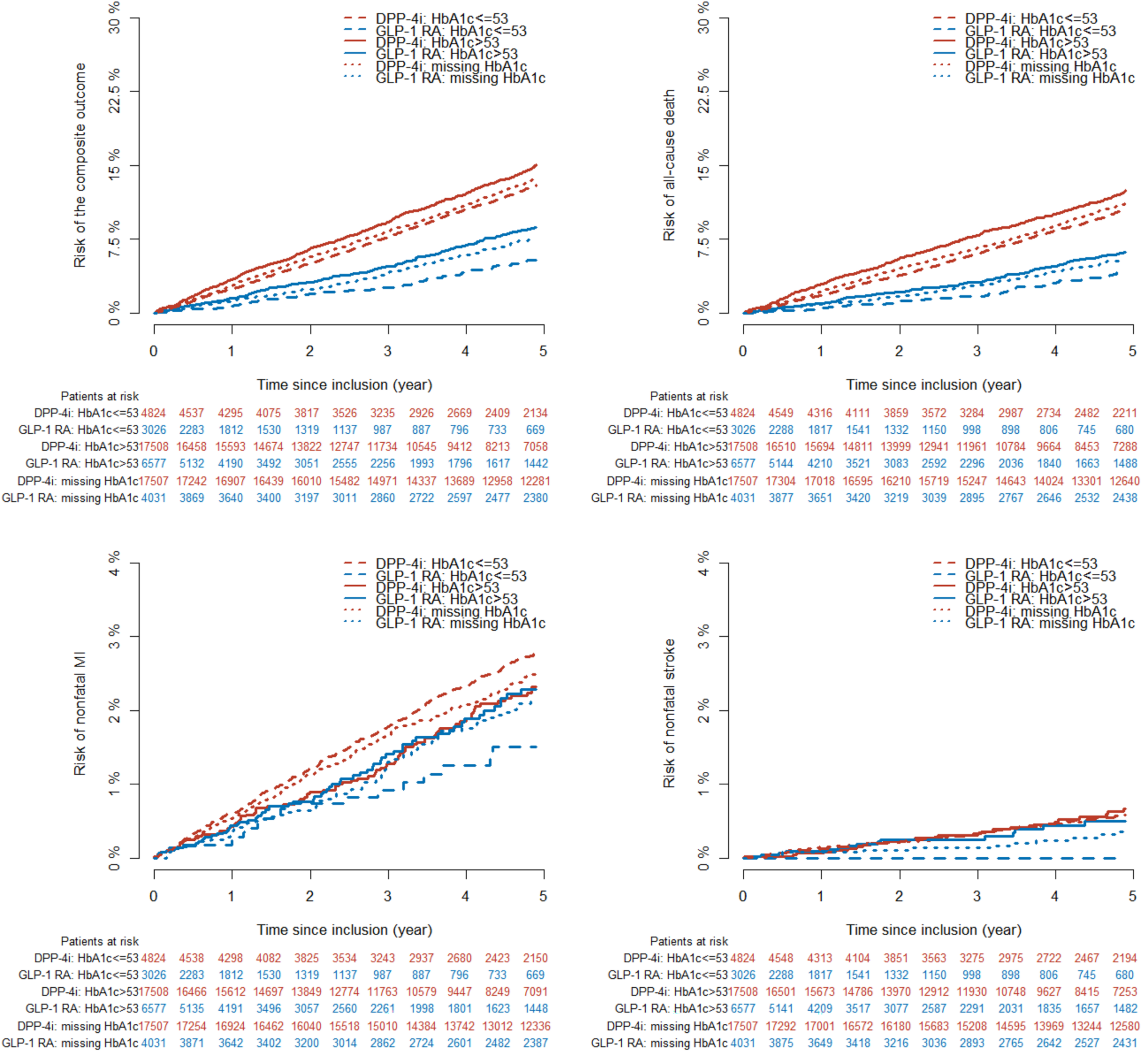
Risk of the composite outcome and components of the composite outcome (nonfatal myocardial infarction, nonfatal stroke, and all‐cause death) according to treatment and achieved HbA1c level (mmol/mol). DPP‐4i, dipeptidyl peptidase‐4 inhibitor; GLP‐1 RA, glucagon‐like‐peptide‐1 receptor agonists; HbA1c, glycated hemoglobin; MI, myocardial infarction.

**FIGURE 3:**
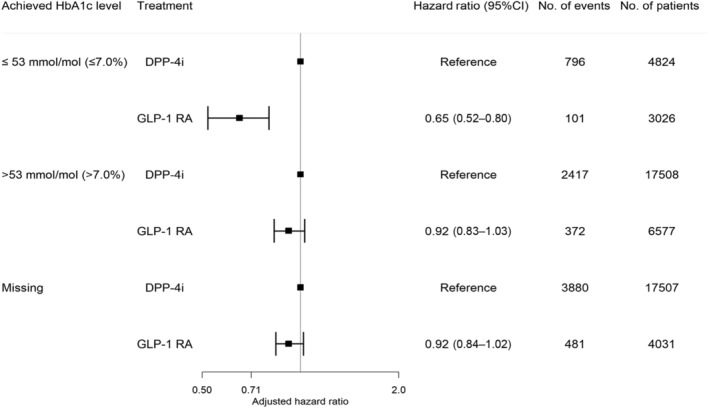
Forest plot of the composite outcome (nonfatal myocardial infarction, non‐fatal stroke, and all‐cause death) according to treatment and achieved HbA1c level (*p* value for interaction <.001). The hazard ratio was adjusted for age, gender, living alone (yes/no), type 2 diabetes duration, pretreatment HbA1c category (≤53 mmol/mol [≤7.0%), >53 mmol/mol (>7.0%], and missing HbA1c), highest achieved educational level, body mass index category (underweight/normal weight, overweight, missing), smoking status (never smoker, never smoker, ex‐smoker, occasional or daily smoker, missing), known comorbidities (atrial fibrillation, cancer, chronic obstructive pulmonary disease, hypertension, chronic kidney disease, cardiovascular disease, heart failure), and inclusion year. CI, confidence interval; DPP‐4i, dipeptidyl peptidase‐4 inhibitor; GLP‐1 RA, glucagon‐like‐peptide‐1 receptor agonists; HbA1c, glycated hemoglobin.

Within a median follow‐up time of 5 years (IQR:2.6–5.0), the following number of events was observed in each achieved HbA1c category among GLP‐1 RA and DPP‐4i users: ≤53 mmol/mol (≤7.0%): 101 (3.3%) versus 796 (16.5%), >53 mmol/mol (>7.0%): 372 (5.7%) versus 2417 (13.8%), missing HbA1c: 481 (11.9%) versus 3880 (22.2%). The 5‐year risk (95% CI) of the composite outcome in GLP1‐RA and DPP4i users according to HbA1c categories was as follows: HbA1c ≤ 53 mmol/mol: 10.3% (8.2–12.3) versus 24.3% (22.7–25.8); HbA1c > 53 mmol/mol: 16.0% (14.3–17.6) versus 21.1% (20.3 –21.9); missing HbA1c: 17.1% (15.7 –18.5) versus 25.6% (24.9 –26.3), as shown in Figure 2. The hazard rate ratio from the multivariable‐adjusted analyses of the composite outcome among GLP‐1 RA users compared to DPP‐4i users across the achieved HbA1c categories was HbA1c ≤ 53 mmol/mol (≤7.0%): 0.65 (0.52–0.80), HbA1c > 53 mmol/mol (>7.0%): 0.92 (0.83–1.03), and missing HbA1c: 0.92 (0.84–1.02) (*p* value for interaction <.001) (Figure 3). The results for the individual components of the composite outcome are shown in Figure S2 in the supplementary material. The outcome of all‐cause death primarily drove the composite outcome. When investigating the association among subgroups, the results remained persistent as in the primary analyses but did not achieve statistical significance for all subgroups (Table S3 in supplementary material).

### 3.3 Subgroup and supplementary analyses

1. When using finer categories of achieved HbA1c levels, we achieved similar results as in the primary analysis, albeit the number of events in each HbA1c category were lower. Tables S4 and S5 in the supplementary material depict the study population's characteristics and analysis results.
2. Using a major adverse cardiovascular outcome that included cardiovascular death instead of all‐cause death did not change the results. Similar 5‐year risk and hazard ratios across different levels of achieved HbA1c categories were observed, as shown in (Table S6 in supplementary material).
3. Among patients with a high adherence to treatment (proportion of days covered >80%) in the 6 months before inclusion, the risk of MACE among patients in treatment with GLP‐1 RA compared to DPP‐4i yielded similar results as in our primary analysis. Lower HbA1c categories were associated with the lowest 5‐year risk and hazard rate of the event (see Table S7 in the supplementary material).
4. The cumulative HbA1c exposure yielded similar results to the primary analyses (Table S8 in the supplementary material).
5. Restricting the cohort to DPP‐4i users included prior to the introduction of GLP‐1 RA in Denmark (2012) yielded similar results to the primary analyses (Table S9 and Figure S3 in the supplementary material). Using sulfonylurea or thiazolidinedione as the active comparator reference group showed similar association as in the primary analyses (Table S10 and Figure S4).

## 4 DISCUSSION

Our observational retrospective cohort study utilizing comprehensive Danish data identified GLP‐1 RA treatment as associated with a cardiovascular outcome reduction compared to DPP‐4i treatment, primarily due to lower all‐cause mortality. The cardiovascular preventive effect associated with GLP‐1 RA compared to DPP‐4i was significantly enhanced when achieving the recommended level of glycemic control, suggesting an interaction between achieving glycemic control and the cardioprotective effect of GLP‐1 RA treatment.

Two meta‐regression analyses have been published to understand whether the cardiovascular benefit associated with GLP‐1 RA in the cardiovascular outcome trials was due to the molecules themselves or the improvement in glycemic control.^11^, ^12^ In both papers, absolute HbA1c reduction was significantly associated with the reduction of MACE, and the regression model indicated a 22% reduction in the risk of MACE per 1% (10.3 mmol/mol) reduction in HbA1c. The conclusion was that the observed MACE benefits in cardiovascular outcome trials partially depended on the decrease in HbA1c. Post‐hoc mediation analysis from the Liraglutide Effect and Action in Diabetes: Evaluation of Cardiovascular Outcome Results (LEADER) trial showed that between 41% and 83% of the total preventive effect of GLP‐1 RA was mediated by the HbA1c reduction.^24^ These results align with this study's results; achieving better glycemic target levels following 6 months of treatment with GLP‐1 RA did improve the cardiovascular preventive benefit. The individual outcome of stroke was characterized by a minimal number of events in each treatment and exposure group, and the results must be interpreted cautiously. But we did find a significant effect of achieving better glycemic control on the outcome of nonfatal stroke compared to the development of nonfatal myocardial infarction. This was also seen in the meta‐regression analyses by Maiorino et al.^12^ The lack of association between HbA1c reduction and the risk of nonfatal myocardial infarction is not entirely understood and needs further investigation. Important to note that the meta‐regression analyses investigated the effect of absolute HbA1c reduction and not the clinical strategy of reaching glycemic target levels, as the trials did not include this particular patient category (ie, achieving target HbA1c levels) and, therefore, could not investigate this. In our study, we investigate this association among a real‐world cohort of GLP‐1 RA users and what is evident from the baseline characteristic of the cohort is that the majority of patients achieving target HbA1c level 6 months following treatment were well regulated (<53 mmol/mol, 7.0%) before treatment. They also exhibited a lower frequency of other cardiovascular risk factors. These findings align with prior Swedish observational data supporting the role of glycemic control and other risk factor management in reducing cardiovascular disease risk.^25^

Both GLP‐1 RA and DPP‐4i enhance the activity of the incretin hormone GLP‐1, but GLP‐1 RAs exhibit a broad impact due to their more potent and sustained GLP‐1 receptor activation.^26^ Notably, GLP‐1 RA induce greater weight loss compared to DPP‐4i and maybe the effect of weight loss and glycemic regulation in the early stages of the treatment (6 months following GLP‐1 RA or DPP‐4i) is associated with a long‐term preventive effect. GLP‐1 RA was associated with a slight reduction in the 5‐year risk of the composite outcome (but not significantly) among those not achieving glycemic control, which aligns with what was observed in the cardiovascular outcome trials underlining the cardiopreventive effects of GLP1‐RA not relating to achieving glycemic control.^24^

Our study benefits from nationwide data completeness and a substantial patient pool. In addition, we used well‐validated measures to assess exposure and outcomes. Yet, a limitation lies in missing HbA1c measurements at 6 months, likely influenced by point‐of‐care testing. Despite this, significant associations were still established. The observational nature prevents precise timing control for HbA1c measurements. In contrast, cardiovascular outcome trials often note the most considerable HbA1c reduction around 3 months after inclusion. To encompass this, we included patients 6 months post‐treatment initiation. The study aimed to investigate the impact of achieving the target HbA1c level within the first 6 months following treatment and not throughout the entire follow‐up period. Therefore, our results are limited to a point‐in‐time assessment and cannot be extrapolated to include information on patients' duration within target glycemic levels. We also investigated the cumulative exposure of HbA1c by using the mean HbA1c value before and after treatment as a way to account for variations in HbA1c level and found the results to be consistent with the main analysis.

Patients in treatment with GLP‐1 RA were more likely to be younger, less comorbid, and receive less concomitant medication at baseline, making them a low‐risk group. Patients with DPP‐4i inhibitors were characterized by older age and higher frequency of comorbidities making them a high‐risk group. We have tried to account for this difference and residual confounding be several sensitivity analyses, but we cannot completely rule out the residual confounding. Notably, our study identifies associations, not causation, with residual confounding due to incomplete information on all possible confounders.

## 5 CONCLUSION

Treatment with GLP‐1 RA was associated with a reduction of a composite outcome of myocardial infarction, stroke, and all‐cause death compared to DPP‐4i treatment when glycemic control was achieved as indicated by an obtained HbA1c level not exceeding 53 mmol/mol. When achieved HbA1c was >53 mmol/mol, GLP‐1 RA was not associated with outcome reduction, suggesting that the cardioprotective effect of GLP‐1 RA may be explained by a combination of glucose lowering and other effects of these drugs.

## AUTHOR CONTRIBUTIONS

Bochra Zareini and Christian Torp‐Pedersen designed the study. Bochra Zareini analyzed the data and drafted the manuscript. Bochra Zareini and Christian Torp‐Pedersen researched data, contributed to discussion, reviewed, and edited the manuscript. Katrine Kold Sørensen, Emil Loldrup Fosbøl, Lars Køber, and Ulrik Pedersen‐Bjergaard contributed to discussion and reviewed and edited the manuscript. All authors approved the final version of the manuscript. Bochra Zareini is the guarantor of this work and, as such, had full access to all the data in the study and takes responsibility for the integrity of the data and the accuracy of the data analysis.

## FUNDING INFORMATION

The research and associated activities were self‐funded by the authors, and no external financial support was received for the development, execution, or completion of this project.

## DISCLOSURES

CTP reports grants from Bayer and Novo Nordisk unrelated to the current study. UP‐B has served on advisory boards for Novo Nordisk, Sanofi, and Vertex and has received lecture fees from Novo Nordisk and Sanofi. LK reports speakers' honorariums from AstraZeneca, Bayer, Boehringer, Novartis, and Novo. EF is the recipient of the independent research funding for valvular heart disease from the Novo Nordisk Foundation. All other authors declare no competing interests.

## Supporting information


**Data S1.** Supporting Information.
