# Supplementary material for: Glucagon‐like‐peptide‐1 receptor agonists versus dipeptidyl peptidase‐4 inhibitors and cardiovascular outcomes in diabetes in relation to achieved glycemic control. A Danish nationwide study
Source: J Diabetes. 2024 May 16;16(6):e13560. doi: 10.1111/1753-0407.13560 (PMC11096807; doi:10.1111/1753-0407.13560)
Supplement: Supplementary file 1 — Data S1. Supporting Information. [file JDB-16-e13560-s001.docx]

**Supplementary material**

**Table S1:** Overview of the details regarding definition of study population, cardiovascular disease, comorbidities and medical treatment.

|  | | **Details** | **ICD-10 and ATC codes used** |
| --- | --- | --- | --- |
| Cardiovascular disease | |  | ICD-10: I |
| Myocardial infarction |  | | ICD-10: I21, I200 |
| Stroke |  | | ICD-10: I60-I64, G45 |
| All-cause death |  | | Any registered date of death |
| Cardiovascular death |  | | Any registered date of death with the underlying cause of death defined as death from cardiovascular disease. |
| Peripheral vascular disease |  | | ICD-10: I70, I739 |
| Heart failure | | Defined from diagnosis codes including heart failure, cardiomyopathies, hypertensive heart failure. | ICD-10: I110, I130, I132, I42, I426-29, I50 |
| Chronic kidney disease | Defined from diagnosis codes of chronic glomerulonephritis, chronic tubulointestinal nephropathy, chronic kidney disease, and diabetic and hypertensive nephropathy. | | ICD-10: E102, E112, E132, E 142, I120, M321B, M300,  M313, M319, N02-N08, N11-N12, N14, N158-N160,  N162-N164, N168, N18-N19, N26, Q612-Q613, Q615, Q619, |
| Atrial fibrillation |  | | ICD-10: I48 |
| Cancer | Defined from all cancer diagnosis codes, excluding non-melanoma skin cancer | | ICD-10: C00-C97 |
| Chronic obstructive pulmonary disease or astma | Baseline COPD or asthma was defined as either a diagnosis with COPD or at least one redemption of drugs for obstructive airway disease within 180 days prior to the index date. | | ICD-10: J42, J44  Inhaled corticosteroids (R03BA, RO3AK06-R03AK12, R03AL08-R03AL09), inhaled short- and long-acting β2-agonists (R03AC, R03AK06-R03AK13, R03AL01-R03AL09), inhaled short- and long-acting muscarinic antagonists (R03BB, R03AL01-R03AL09) |
| Hypertension | Defined from combination  treatment with a least two classes of antihypertensive drugs  (Adrenergic α-antagonist, nonloop-  diuretics, vasodilators, betablockers,  calcium channel  blockers, and renin-angiotensin  system inhibitors) | | ATC: C02A, C02B, C02C, C02DA,  C02DB, C02DD, C02DG, C02L, C03A, C03B, C03D, C03E, C03X,  C07A, C07B, C07C, C07D, C07F,  C08, C09AA, C09BA, C09BB,  C09CA, C09DA, C09DB, C09XA02,  C09XA52 |
| **Pharmacotherapy** | | | |
| Statins | |  | ATC: C10A |
| Beta-blockers | |  | ATC: C07 |
| Mineralocorticoid receptor antagonists | |  | ATC: CO3D |
| ADP | |  | ATC: B01AC04, B01AC06, B01AC22, B01AC24, B01AC25, NO2BA01 |
| NOAC’s | |  | ATC: B01AF01, B01AF02, B01AE07 |
| Vitamin K antagonists | |  | ATC: B01AA03-04 |
| Ca antagonists | |  | ATC: C08 |
| Digoxin | |  | ATC: C01AA05 |
| Thiazide | |  | ATC CO3A |
| Renin angiotensin system inhibitors | | Including: angiotensin-converting-enzyme inhibitors, angiotensin-II receptor blockers | ATC: C09 |
| Loop diuretics | |  | ATC: C03CA01 |
| Insulin | |  | ATC: A10A |
| Metformin | |  | ATC: A10BA02 |
| Sulfonylurea | |  | ATC: A10BB |
| Thiazolidinedione | |  | ATC: A10BG |
| DDP-4 inhibitor | |  | ATC A10BH |
| GLP-1 RA | |  | ATC: A10BJ |
| SGLT2 inhibitor | |  | ATC: A10BK |
| Combination of two glucose lowering agents. | |  | ATC: A10BD |

Abbreviations: ICD: International classification of disease, ATC: anatomical therapeutic code, ACE: angiotensin converting enzyme, ARB: angiotensin II receptor blocker, ADP: adenin diphosphate receptor, MRA mineralocorticoid receptor antagonists, DPP-4 dipeptidyl peptidase-4, GLP-1 glucagon-like peptide-1, SGLT2 sodium-glucose-cotransporter-2.

**Table S2**: Overview of the definitions of the conditions that would make glycated haemoglobin invalid as a monitoring measure

| **Conditions disqualifying HbA1c as monitoring measure** | | |
| --- | --- | --- |
| Severe liver disease/splenomegaly |  | ICD-10: B150, B160, B162, B190, I85, K703, K704, K717, K72, K743-K746, K766, I982, R16, D709E, K768B, Q890C, Z944 |
| Alcoholism |  | ICD-10: E244, E529A, F10, G312, G621, I426, K70, K852, K860, L278A, T51, Z714, Z721. |
| B12 deficiency | Registered diagnoses code or registered hospital administered B12 supplementation 180 days prior to inclusion date. | ICD-10: D51, E538D  Administration: BOHC2 |
| Iron deficiency | Registered diagnoses code or registered hospital administered iron supplementation 180 days prior to inclusion date. | ICD-10: D51, E538D  Administration: BOHC1 |
| Haemoglobinopathies/haemolytic anaemia |  | ICD-10: D56-D59 |
| End-stage renal disease | Defined from a diagnosis code or procedural codes. | ICD-10: N185, Z992, Z940, T856C  Procedural codes: KKAS, KYK, KJAK14, KJAK13,BJFZ, BJFD |
| Use of certain drugs |  | ATC codes:  Trimethoprim: J01EA  Sulfamethoxazole: J01EE  Sulfasalazine: A07EC01  Hydroxyurea: L01XX05  Dapsone: J04BA02 |

| **Subgroup analyses** |  | **N events** | **N persons** | **5-year risk (95% CI)** | **Univariate hazard ratio (95% CI)** | **Adjusted hazard ratio (95% CI)*** | **p-value for interaction** |
| --- | --- | --- | --- | --- | --- | --- | --- |
| **Among females** |  |  |  |  |  |  |  |
| HbA1c ≤ 53 mmol/mol (≤ 7.0%) | DPP-4i | 319 | 2277 | 20.7 (18.6;22.9) | Reference | Reference | 0.57 |
|  | GLP-1 RA | 56 | 1806 | 8.8 (6.4;11.2) | 0.37 (0.28;0.50) | 0.73 (0.55;0.98) |  |
| HbA1c >53 mmol/mol (>7.0%) | DPP-4i | 833 | 6477 | 20.0 (18.7;21.3) | Reference | Reference |  |
|  | GLP-1 RA | 119 | 2679 | 13.6 (11.1;16.1) | 0.58 (0.47;0.70) | 0.88 (0.72;1.07) |  |
| Missing HbA1c information | DPP-4i | 1374 | 6786 | 23.4 (22.3;24.5) | Reference | Reference |  |
|  | GLP-1 RA | 166 | 1922 | 12.7 (10.9;14.6) | 0.50 (0.42;0.58) | 0.87 (0.73;1.02) |  |
|  |  |  |  |  |  |  |  |
| **Among males** |  |  |  |  |  |  |  |
| HbA1c ≤ 53 mmol/mol (≤ 7.0%) | DPP-4i | 477 | 2547 | 27.4 (25.2;29.6) | Reference | Reference | 0.004 |
|  | GLP-1 RA | 45 | 1220 | 12.8 (9.0;16.6) | 0.38 (0.28;0.52) | 0.57 (0.42;0.77) |  |
| HbA1c >53 mmol/mol (>7.0%) | DPP-4i | 1584 | 11031 | 21.7 (20.7;22.7) | Reference | Reference |  |
|  | GLP-1 RA | 253 | 3898 | 17.5 (15.4;19.7) | 0.75 (0.66;0.86) | 0.94 (0.82;1.08) |  |
| Missing HbA1c information | DPP-4i | 2506 | 10721 | 27.0 (26.1;27.9) | Reference | Reference |  |
|  | GLP-1 RA | 315 | 2109 | 20.9 (18.8;23.0) | 0.72 (0.64;0.81) | 0.96 (0.85;1.08) |  |
|  |  |  |  |  |  |  |  |
| **Among patients free of CVD at inclusion** |  |  |  |  |  |  |  |
| HbA1c ≤ 53 mmol/mol (≤ 7.0%) | DPP-4i | 456 | 3307 | 20.7 (18.9;22.4) | Reference | Reference | 0.17 |
|  | GLP-1 RA | 58 | 2119 | 8.8 (6.5;11.1) | 0.36 (0.28;0.48) | 0.69 (0.52;0.90) |  |
| HbA1c >53 mmol/mol (>7.0%) | DPP-4i | 1449 | 12369 | 18.4 (17.5;19.3) | Reference | Reference |  |
|  | GLP-1 RA | 202 | 4665 | 13.3 (11.5;15.2) | 0.64 (0.55;0.74) | 0.89 (0.76;1.03) |  |
| Missing HbA1c information | DPP-4i | 2337 | 12284 | 22.3 (21.5;23.1) | Reference | Reference |  |
|  | GLP-1 RA | 280 | 2857 | 14.5 (12.9;16.1) | 0.61 (0.54;0.68) | 0.91 (0.80;1.03) |  |
|  |  |  |  |  |  |  |  |
| **Among patients with CVD at inclusion** |  |  |  |  |  |  |  |
| HbA1c < 53 mmol/mol (≤ 7.0%) | DPP-4i | 340 | 1517 | 31.8 (28.9;34.8) | Reference | Reference | 0.015 |
|  | GLP-1 RA | 43 | 907 | 13.6 (9.4;17.7) | 0.36 (0.26;0.49) | 0.59 (0.43;0.81) |  |
| HbA1c >53 mmol/mol (>7.0%) | DPP-4i | 968 | 5139 | 27.4 (25.8;29.0) | Reference | Reference |  |
|  | GLP-1 RA | 170 | 1912 | 21.3 (18.2;24.4) | 0.71 (0.61;0.84) | 0.97 (0.82;1.14) |  |
| Missing HbA1c information | DPP-4i | 1543 | 5223 | 33.2 (31.9;34.6) | Reference | Reference |  |
|  | GLP-1 RA | 201 | 1174 | 23.0 (20.2;25.8) | 0.63 (0.54;0.73) | 0.93 (0.80;1.08) |  |
|  |  |  |  |  |  |  |  |
| **Among patients age 0-60y** |  |  |  |  |  |  |  |
| HbA1c ≤ 53 mmol/mol (≤ 7.0%) | DPP-4i | 70 | 1407 | 7.7 (5.9;9.4) | Reference | Reference | 0.52 |
|  | GLP-1 RA | 38 | 1750 | 6.0 (4.0;7.9) | 0.86 (0.57:1.35) | 0.87 (0.51;1.12) |  |
| HbA1c >53 mmol/mol (>7.0%) | DPP-4i | 394 | 7151 | 8.4 (7.5;9.2) | Reference | Reference |  |
|  | GLP-1 RA | 137 | 3901 | 9.3 (7.7;10.9) | 1.11 (0.91;1.35) | 1.02 (0.82;1.21) |  |
| Missing HbA1c information | DPP-4i | 690 | 7166 | 11.2 (10.4;12.0) | Reference | Reference |  |
|  | GLP-1 RA | 189 | 2493 | 10.7 (9.2:12.2) | 0.93 (0.79;1.09) | 1.03 (0.85;1.17) |  |
|  |  |  |  |  |  |  |  |
| **Among patients age 61-80y** |  |  |  |  |  |  |  |
| HbA1c ≤ 53 mmol/mol (≤ 7.0%) | DPP-4i | 516 | 2912 | 25.2 (24.2;28.3) | Reference | Reference | 0.007 |
|  | GLP-1 RA | 54 | 1226 | 16.4 (12.0;20.8) | 0.53 (0.40;0.71) | 0.54 (0.41;0.71) |  |
| HbA1c >53 mmol/mol (>7.0%) | DPP-4i | 1452 | 8884 | 26.0 (24.7;27.2) | Reference | Reference |  |
|  | GLP-1 RA | 203 | 2513 | 26.8 (23.3;30.3) | 0.93 (0.80;1.07) | 0.87 (0.75;1.00) |  |
| Missing HbA1c information | DPP4i | 2422 | 9120 | 30.6 (29.6;31.7) | Reference | Reference |  |
|  | GLP-1 RA | 270 | 1487 | 27.1 (24.3;29.9) | 0.84 (0.74;0.95) | 0.84 (0.74;0.95) |  |
|  |  |  |  |  |  |  |  |
| **Among patients age >80y** |  |  |  |  |  |  |  |
| HbA1c ≤ 53 mmol/mol (≤ 7.0%) | DPP-4i | 210 | 505 | 67.4 (61.3;73.4) | Reference | Reference | 0.99 |
|  | GLP-1 RA | 9 | 50 | 100 | 0.94 (0.48:1.83) | 0.90  (0.44;1.67) |  |
| HbA1c >53 mmol/mol (>7.0%) | DPP-4i | 571 | 1473 | 67.8 (64.0:71.5) | Reference | Reference |  |
|  | GLP-1 RA | 32 | 163 | 58.5 (41.0;76.1) | 1.09 (0.87;1.51) | 0.96 (0.73;1.43) |  |
| Missing HbA1c information | DPP-4i | 768 | 1221 | 77.1 (74.3;79.8) | Reference | Reference |  |
|  | GLP-1 RA | 22 | 51 | 72.0 (54.8;89.2) | 0.90 (0.62;1.32) | 0.94 (0.64;1.38) |  |
| **Among patients included between 2007-2011** |  |  |  |  |  |  |  |
| HbA1c ≤ 53 mmol/mol (≤ 7.0%) | DPP-4i | 138 | 394 | 35.1 (30.4;39.8) | Reference | Reference | 0.62 |
|  | GLP-1 RA | 20 | 173 | 11.6 (6.8;16.8) | 0.28 (0.18;0.45) | 0.52 (0.32;0.83) |  |
| HbA1c >53 mmol/mol (>7.0%) | DPP-4i | 352 | 1186 | 29.7(27.1;32.3) | Reference | Reference |  |
|  | GLP-1 RA | 86 | 386 | 22.3 (18.1;26.4) | 0.71 (0.56;0.90) | 0.85 (0.67;1.08) |  |
| Missing HbA1c information | DPP-4i | 1802 | 5687 | 31.7 (30.5;33.0) | Reference | Reference |  |
|  | GLP-1 RA | 230 | 1033 | 22.3 (19.8;24.9) | 0.66 (0.58;0.76) | 0.90 (0.78;1.03) |  |
| **Among patients included between 2012-2016** |  |  |  |  |  |  |  |
| HbA1c ≤ 53 mmol/mol (≤ 7.0%) | DPP-4i | 439 | 2067 | 21.3 (19.5;23.0) | Reference | Reference | 0.26 |
|  | GLP-1 RA | 47 | 523 | 9.0 (6.5;11.5) | 0.39 (0.29;0.53) | 0.75 (0.55;1.02) |  |
| HbA1c >53 mmol/mol (>7.0%) | DPP-4i | 1294 | 6809 | 19.0 (18.1;20.0) | Reference | Reference |  |
|  | GLP-1 RA | 160 | 1175 | 13.6 (11.7;15.6) | 0.69 (0.58;0.81) | 0.99 (0.84;1.17) |  |
| Missing HbA1c information | DPP-4i | 1744 | 8314 | 21.0 (20.1;21.9) | Reference | Reference |  |
|  | GLP-1 RA | 206 | 1535 | 13.4 (11.7;15.2) | 0.61 (0.53;0.70) | 0.99 (0.79;1.06) |  |
| **Among patients included between 2017-2021** |  |  |  |  |  |  |  |
| HbA1c ≤ 53 mmol/mol (≤ 7.0%) | DPP-4i | 219 | 2363 | 17.9 (14.0;21.8) | Reference | Reference | 0.16 |
|  | GLP-1 RA | 34 | 2330 | 6.6 (2.3;10.8) | 0.30 (0.21;0.43) | 0.65 (0.45;0.93) |  |
| HbA1c >53 mmol/mol (>7.0%) | DPP-4i | 771 | 9513 | 16.7 (14.2;19.1) | Reference | Reference |  |
|  | GLP-1 RA | 126 | 5016 | 8.7 (6.3;11.1) | 0.57 (0.47;0.69) | 0.95 (0.78;1.15) |  |
| Missing HbA1c information | DPP-4i | 334 | 3506 | 14.9 (12.9;16.8) | Reference | Reference |  |
|  | GLP-1 RA | 45 | 1463 | 10.9 (6.2;15.5) | 0.48 (0.35;0.66) | 0.83 (0.61;1.14) |  |
|  |  |  |  |  |  |  |  |

**Table S3:** Risk of the composite outcome according to treatment and achieved HbA1c level in specific subgroups. The composite outcome included non-fatal myocardial infarction, non-fatal stroke and all-cause death. * The model was adjusted for the following covariates: age, gender, living alone (yes/no), type 2 diabetes duration, pre-treatment HbA1c category (≤ 53 mmol/mol (≤ 7.0%), >53 mmol/mol (> 7.0%), and missing HbA1c), highest achieved educational level, body mass index category (underweight/normal weight, overweight, missing), smoking status (never smoker, never smoker, ex-smoker, occasional or daily smoker, missing), known comorbidities (atrial fibrillation, cancer, chronic obstructive pulmonary disease, hypertension, chronic kidney disease, cardiovascular disease, heart failure), and inclusion year. Abbreviations: n: number, CI: confidence interval, HbA1c: glycated hemoglobin, DPP-4i: dipeptidyl peptidase-4 inhibitor, GLP-1 RA: glucagon-like-peptide-1 receptor agonists.

|  |  | **Missing HbA1c** | | **HbA1c <48 mmol/mol**  **(< 6.5%)** | | **HbA1c 48-53 mmol/mol**  **(6.5-7.0%)** | | **HbA1c 54-58 mmol/mol**  **(7.1-7.5%)** | | **HbA1c > 58 mmol/mol**  **(>7.5%)** | |
| --- | --- | --- | --- | --- | --- | --- | --- | --- | --- | --- | --- |
|  |  | **GLP-1 RA (n=4031)** | **DPP-4i (n=1750)** | **GLP-1 RA (n=1554)** | **DPP-4i (n=1523)** | **GLP-1 RA (n=1472)** | **DPP-4i (n=3301)** | **GLP-1 RA (n=1603)** | **DPP-4i (n=4554)** | **GLP-1 RA (n=4974)** | **DPP-4i (n=12954)** |
| Age | median (iqr) | 56 (47.4, 64.8) | 62.8 (54.1, 70.8) | 55.3 (43.2, 64.6) | 68.9 (60.5, 76.2) | 59.7 (51.8, 67.6) | 66.3 (57.5, 73.6) | 59.2 (51.3, 67.3) | 65.5 (56.6, 73.1) | 56.5 (48.6, 64.8) | 62.2 (53.5, 71.4) |
| Males |  | 2109 (52.3) | 10721 (61.2) | 540 (34.7) | 772 (50.7) | 680 (46.2) | 1775 (53.8) | 815 (50.8) | 2622 (57.6) | 3083 (62.0) | 8409 (64.9) |
| T2D duration (years) | median (iqr) | 2.9 (1.3, 5.4) | 2.9 (1.1, 5.3) | 2.5 (1.2, 5.6) | 2.8 (1.1, 5.7) | 2.8 (1.1, 5.8) | 3.5 (1.5, 6.3) | 3.5 (1.7, 6.3) | 4 (1.9, 6.7) | 3 (1.0, 6.1) | 3.7 (1.2, 6.6) |
| Pre-treatment HbA1c | ≤53 mmol/mol (≤ 7.0%) | 797 (19.8) | 1,686 (9.6) | 956 (61.5) | 992 (65.1) | 1,036 (70.4) | 2,082 (63.1) | 1,055 (65.8) | 2,456 (53.9) | 2,257 (45.4) | 4,053 (31.3) |
|  | >53 mmol/mol (> 7.0%) | 329 (8.2) | 1302 (7.4) | 8 (0.5) | 68 (4.5) | 48 (3.3) | 310 (9.4) | 143 (8.9) | 942 (20.7) | 1450 (29.2) | 5732 (44.2) |
|  | missing | 2905 (72.1) | 14519 (82.9) | 590 (38.0) | 463 (30.4) | 388 (26.4) | 909 (27.5) | 405 (25.3) | 1156 (25.4) | 1267 (25.5) | 3169 (24.5) |
| Highest attained education | Basic Education | 1,435 (35.6) | 7,369 (42.1) | 428 (27.5) | 611 (40.1) | 491 (33.4) | 1,249 (37.8) | 514 (32.1) | 1,717 (37.7) | 1,737 (34.9) | 5,165 (39.9) |
|  | General upper secondary education | 1797 (44.6) | 7422 (42.4) | 706 (45.4) | 624 (41.0) | 656 (44.6) | 1462 (44.3) | 767 (47.8) | 1992 (43.7) | 2300 (46.2) | 5598 (43.2) |
|  | Bachelor level education | 637 (15.8) | 2191 (12.5) | 330 (21.2) | 212 (13.9) | 258 (17.5) | 474 (14.4) | 265 (16.5) | 670 (14.7) | 770 (15.5) | 1731 (13.4) |
|  | Masters or PhD | 162 (4.0) | 525 (3.0) | 90 (5.8) | 76 (5.0) | 67 (4.6) | 116 (3.5) | 57 (3.6) | 175 (3.8) | 167 (3.4) | 460 (3.6) |
| Living alone |  | 829 (20.6) | 4491 (25.7) | 136 (8.8) | 270 (17.7) | 85 (5.8) | 466 (14.1) | 98 (6.1) | 591 (13.0) | 388 (7.8) | 1647 (12.7) |
| BMI category | Underwight/Normal weight | 26 (0.6) | 267 (1.5) | 9 (0.6) | 93 (6.1) | 24 (1.6) | 113 (3.4) | 33 (2.1) | 177 (3.9) | 69 (1.4) | 388 (3.0) |
|  | Overweight | 879 (21.8) | 2166 (12.4) | 421 (27.1) | 305 (20.0) | 522 (35.5) | 814 (24.7) | 622 (38.8) | 1250 (27.4) | 1752 (35.2) | 3108 (24.0) |
|  | Missing | 3126 (77.5) | 15074 (86.1) | 1124 (72.3) | 1125 (73.9) | 926 (62.9) | 2374 (71.9) | 948 (59.1) | 3127 (68.7) | 3153 (63.4) | 9458 (73.0) |
| Smoking status | Never smoker | 742 (18.4) | 2,984 (17.0) | 192 (12.4) | 307 (20.2) | 258 (17.5) | 715 (21.7) | 311 (19.4) | 1,019 (22.4) | 879 (17.7) | 2,414 (18.6) |
|  | Ex smoker | 523 (13.0) | 1897 (10.8) | 192 (12.4) | 188 (12.3) | 224 (15.2) | 405 (12.3) | 275 (17.2) | 611 (13.4) | 682 (13.7) | 1519 (11.7) |
|  | Occasional or daily smoker | 343 (8.5) | 1315 (7.5) | 83 (5.3) | 115 (7.6) | 109 (7.4) | 249 (7.5) | 129 (8.0) | 371 (8.1) | 457 (9.2) | 1015 (7.8) |
|  | Missing | 2423 (60.1) | 11311 (64.6) | 1087 (69.9) | 913 (59.9) | 881 (59.9) | 1932 (58.5) | 888 (55.4) | 2553 (56.1) | 2956 (59.4) | 8006 (61.8) |
| e-GFR ml/min/1.73m2 | 30-59 | 48 (1.2) | 361 (2.1) | 48 (3.1) | 201 (13.2) | 85 (5.8) | 343 (10.4) | 94 (5.9) | 478 (10.5) | 196 (3.9) | 1,083 (8.4) |
|  | 60-89 | 190 (4.7) | 739 (4.2) | 215 (13.8) | 255 (16.7) | 234 (15.9) | 671 (20.3) | 245 (15.3) | 1021 (22.4) | 730 (14.7) | 2622 (20.2) |
|  | >90 | 166 (4.1) | 463 (2.6) | 170 (10.9) | 125 (8.2) | 184 (12.5) | 364 (11.0) | 226 (14.1) | 538 (11.8) | 852 (17.1) | 2082 (16.1) |
|  | missing | 3627 (90.0) | 15944 (91.1) | 1121 (72.1) | 942 (61.9) | 969 (65.8) | 1923 (58.3) | 1038 (64.8) | 2517 (55.3) | 3196 (64.3) | 7167 (55.3) |
| LDL mmol/L | 0-2.5 | 253 (6.3) | 745 (4.3) | 328 (21.1) | 361 (23.7) | 353 (24.0) | 838 (25.4) | 354 (22.1) | 1,212 (26.6) | 1,134 (22.8) | 3,435 (26.5) |
|  | >2.5 | 109 (2.7) | 291 (1.7) | 138 (8.9) | 161 (10.6) | 109 (7.4) | 281 (8.5) | 101 (6.3) | 344 (7.6) | 401 (8.1) | 1190 (9.2) |
|  | missing | 3669 (91.0) | 16471 (94.1) | 1088 (70.0) | 1001 (65.7) | 1010 (68.6) | 2182 (66.1) | 1148 (71.6) | 2998 (65.8) | 3439 (69.1) | 8329 (64.3) |
| Hypertension |  | 1454 (36.1) | 6760 (38.6) | 447 (28.8) | 528 (34.7) | 496 (33.7) | 1159 (35.1) | 561 (35.0) | 1598 (35.1) | 1621 (32.6) | 4273 (33.0) |
| Atrial fibrillation |  | 186 (4.6) | 1064 (6.1) | 78 (5.0) | 144 (9.5) | 90 (6.1) | 220 (6.7) | 96 (6.0) | 345 (7.6) | 271 (5.4) | 862 (6.7) |
| Cancer |  | 214 (5.3) | 1286 (7.3) | 76 (4.9) | 179 (11.8) | 107 (7.3) | 318 (9.6) | 118 (7.4) | 411 (9.0) | 283 (5.7) | 1087 (8.4) |
| COPD |  | 625 (15.5) | 2368 (13.5) | 250 (16.1) | 247 (16.2) | 240 (16.3) | 454 (13.8) | 232 (14.5) | 589 (12.9) | 660 (13.3) | 1643 (12.7) |
| CKD |  | 114 (2.8) | 634 (3.6) | 41 (2.6) | 224 (14.7) | 41 (2.8) | 212 (6.4) | 64 (4.0) | 177 (3.9) | 174 (3.5) | 471 (3.6) |
| Cardiovascular disease |  | 1108 (27.5) | 4872 (27.8) | 417 (26.8) | 471 (30.9) | 437 (29.7) | 891 (27.0) | 466 (29.1) | 1245 (27.3) | 1350 (27.1) | 3502 (27.0) |
| Stroke |  | 151 (3.7) | 878 (5.0) | 50 (3.2) | 107 (7.0) | 53 (3.6) | 173 (5.2) | 65 (4.1) | 250 (5.5) | 219 (4.4) | 687 (5.3) |
| Heart failure |  | 171 (4.2) | 769 (4.4) | 49 (3.2) | 130 (8.5) | 69 (4.7) | 183 (5.5) | 54 (3.4) | 209 (4.6) | 193 (3.9) | 630 (4.9) |
| PAD |  | 101 (2.5) | 609 (3.5) | 24 (1.5) | 74 (4.9) | 38 (2.6) | 111 (3.4) | 42 (2.6) | 151 (3.3) | 103 (2.1) | 405 (3.1) |
| ASA |  | 1044 (25.9) | 5425 (31.0) | 226 (14.5) | 408 (26.8) | 286 (19.4) | 828 (25.1) | 333 (20.8) | 1080 (23.7) | 864 (17.4) | 2925 (22.6) |
| Statins |  | 2576 (63.9) | 12393 (70.8) | 817 (52.6) | 1036 (68.0) | 1029 (69.9) | 2400 (72.7) | 1159 (72.3) | 3383 (74.3) | 3207 (64.5) | 8796 (67.9) |
| ACE/ARB |  | 2381 (59.1) | 10955 (62.6) | 764 (49.2) | 903 (59.3) | 952 (64.7) | 2046 (62.0) | 1026 (64.0) | 2876 (63.2) | 2970 (59.7) | 7659 (59.1) |
| Betablockers |  | 951 (23.6) | 4394 (25.1) | 285 (18.3) | 478 (31.4) | 356 (24.2) | 915 (27.7) | 384 (24.0) | 1126 (24.7) | 1050 (21.1) | 3137 (24.2) |
| Ca channel blockers |  | 1005 (24.9) | 4882 (27.9) | 344 (22.1) | 473 (31.1) | 435 (29.6) | 979 (29.7) | 441 (27.5) | 1283 (28.2) | 1336 (26.9) | 3287 (25.4) |
| Thiazide |  | 599 (14.9) | 2483 (14.2) | 214 (13.8) | 197 (12.9) | 245 (16.6) | 495 (15.0) | 261 (16.3) | 628 (13.8) | 674 (13.6) | 1552 (12.0) |
| MRA |  | 216 (5.4) | 641 (3.7) | 103 (6.6) | 88 (5.8) | 95 (6.5) | 188 (5.7) | 78 (4.9) | 204 (4.5) | 251 (5.0) | 514 (4.0) |
| Digoxin |  | 66 (1.6) | 610 (3.5) | 8 (0.5) | 62 (4.1) | 19 (1.3) | 109 (3.3) | 30 (1.9) | 155 (3.4) | 121 (2.4) | 484 (3.7) |
| Loop diuretics |  | 458 (11.4) | 2080 (11.9) | 181 (11.6) | 322 (21.1) | 197 (13.4) | 462 (14.0) | 187 (11.7) | 529 (11.6) | 506 (10.2) | 1401 (10.8) |
| ADPi |  | 1131 (28.1) | 5938 (33.9) | 276 (17.8) | 503 (33.0) | 352 (23.9) | 979 (29.7) | 401 (25.0) | 1297 (28.5) | 1069 (21.5) | 3557 (27.5) |
| Vitamin K antagonist |  | 148 (3.7) | 915 (5.2) | 40 (2.6) | 98 (6.4) | 38 (2.6) | 151 (4.6) | 44 (2.7) | 234 (5.1) | 120 (2.4) | 534 (4.1) |
| NOAC's |  | 75 (1.9) | 248 (1.4) | 58 (3.7) | 94 (6.2) | 79 (5.4) | 136 (4.1) | 81 (5.1) | 204 (4.5) | 223 (4.5) | 505 (3.9) |

**Table S4:** Characteristics of the study population at time of inclusion. Abbreviations: n: number, IQR: interquartile range, T2D: type 2 diabetes, HbA1c: glycated haemoglobin, BMI: body mass index, e-GFR: estimated glomerular filtration rate, LDL: low-density lipoprotein, PAD: peripheral artery disease, COPD: chronic obstructive pulmonary disease, CKD: chronic kidney disease, ACE: angiotensin converting en-zyme, ARB: angiotensin II receptor blocker, ADP: adenin diphosphate receptor, NOAC’s: new oral anticoagulants, MRA mineralocorticoid receptor antagonists, DPP-4 dipeptidyl peptidase-4, GLP-1 glucagon-like peptide-1.

|  |  | **N events** | **N persons** | **5-year risk (95% CI)** | **Univariate hazard ratio (95% CI)** | **Adjusted hazard ratio (95% CI)*** | **P-value for interaction** |
| --- | --- | --- | --- | --- | --- | --- | --- |
| **Achieved HbA1c category** |  |  |  |  |  |  |  |
| HbA1c < 48 mmol/mol (< 6.5%) | DPP4i | 319 | 1523 | 28.9 (26.1;31.8) | Reference | Reference | <0.001 |
|  | GLP-1 RA | 51 | 1554 | 8.6 (6.2;11.1) | 0.25 (0.19;0.34) | 0.59 (0.43;0.78) |  |
| HbA1c 48-53 mmol/mol (6.5-7.0%) | DPP4i | 477 | 3301 | 21.9 (20.1;23.8) | Reference | Reference |  |
|  | GLP-1 RA | 50 | 1472 | 12.7 (9.1;16.2) | 0.48 (0.36:0.64) | 0.70 (0.52;0.94) |  |
| HbA1c 54-58 mmol/mol (7.1-7.5%) | DPP4i | 596 | 4554 | 20.6 (19.1;22.2) | Reference | Reference |  |
|  | GLP-1 RA | 70 | 1603 | 15.1 (11.6;18.6) | 0.61 (0.47;0.78) | 0.83 (0.65;1.06) |  |
| HbA1c > 58 mmol/mol (>7.5%) | DPP4i | 1821 | 12954 | 21.0 (20.1;21.9) | Reference | Reference |  |
|  | GLP-1 RA | 302 | 4974 | 16.2 (14.4;18.1) | 0.70 (0.62;0.79) | 0.94 (0.83;1.06) |  |
| Missing HbA1c information | DPP4i | 3880 | 17507 | 25.6 (24.9;26.3) | Reference | Reference |  |
|  | GLP-1 RA | 481 | 4031 | 17.1 (15.7;18.5) | 0.62 (0.56;0.68) | 0.92 (0.84;1.02) |  |

**Table S5:** Risk of the composite outcome according to treatment and achieved level of HbA1c. The composite outcome included non-fatal myocardial infarction, non-fatal stroke and all-cause death. * The model was adjusted for the following covariates: age, gender, living alone (yes/no), type 2 diabetes duration, pre-treatment HbA1c category (≤ 53 mmol/mol (≤ 7.0%), >53 mmol/mol (> 7.0%), and missing HbA1c), highest achieved educational level, body mass index category (underweight/normal weight, overweight, missing), smoking status (never smoker, never smoker, ex-smoker, occasional or daily smoker, missing), known comorbidities (atrial fibrillation, cancer, chronic obstructive pulmonary disease, hypertension, chronic kidney disease, cardiovascular disease, heart failure), and inclusion year. Abbreviations: n: number, CI: confidence interval, HbA1c: glycated hemoglobin, DPP-4i: dipeptidyl peptidase-4 inhibitor, GLP-1 RA: glucagon-like-peptide-1 receptor agonist.

|  |  | **N events** | **N persons** | **5-year risk (95% CI)** | **Univariate hazard ratio (95% CI)** | **Adjusted hazard ratio (95% CI)*** | **P value for interaction** |
| --- | --- | --- | --- | --- | --- | --- | --- |
| **Achieved HbA1c level** |  |  |  |  |  |  |  |
| HbA1c ≤ 53 mmol/mol (≤7.0%) | DPP4i | 216 | 3529 | 11.2 (9.6;12.7) | Reference | Reference | 0.122 |
|  | GLP-1 RA | 25 | 1003 | 4.3 (2.6;6.0) | 0.35 (0.23;0.52) | 0.67 (0.43;1.03) |  |
| HbA1c >53 mmol/mol (> 7.0%) | DPP4i | 657 | 12683 | 10.7 (9.8;11.6) | Reference | Reference |  |
|  | GLP-1 RA | 112 | 2343 | 9.1 (7.4;10.8) | 0.77 (0.63;0.94) | 0.91 (0.72:1.15) |  |
| Missing HbA1c information | DPP4i | 1453 | 16159 | 12.3 (11.7;12.9) | Reference | Reference |  |
|  | GLP-1 RA | 173 | 2970 | 7.7 (6.6;8.8) | 0.60 (0.52;0.71) | 0.91 (0.75;1.10) |  |

**Table S6:** Risk of the composite outcome according to treatment and achieved level of HbA1c. The composite outcome included non-fatal myocardial infarction, non-fatal stroke and cardiovascular death. * The model was adjusted for the following covariates: age, gender, living alone (yes/no), type 2 diabetes duration, pre-treatment HbA1c category (≤ 53 mmol/mol (≤ 7.0%), >53 mmol/mol (> 7.0%), and missing HbA1c), highest achieved educational level, body mass index category (underweight/normal weight, overweight, missing), smoking status (never smoker, never smoker, ex-smoker, occasional or daily smoker, missing), known comorbidities (atrial fibrillation, cancer, chronic obstructive pulmonary disease, hypertension, chronic kidney disease, cardiovascular disease, heart failure), and inclusion year. Abbreviations: n: number, CI: confidence interval, HbA1c: glycated hemoglobin, DPP-4i: dipeptidyl peptidase-4 inhibitor, GLP-1 RA: glucagon-like-peptide-1 receptor agonist.

|  |  | **N events** | **N persons** | **5-year risk (95% CI)** | **Univariate hazard ratio (95% CI)** | **Adjusted hazard ratio (95% CI)*** | **P value for interaction** |
| --- | --- | --- | --- | --- | --- | --- | --- |
| **Achieved HbA1c category** | |  |  |  |  |  | 0.36 |
| HbA1c ≤ 53 mmol/mol (≤7.0%) | DPP-4i | 485 | 3410 | 20.5 (18.8;22.2) | Reference | Reference |  |
|  | GLP-1 RA | 86 | 2281 | 10.4 (8.2;12.7) | 0.48 (0.38;0.61) | 0.77 (0.61;0.97) |  |
| HbA1c >53 mmol/mol (> 7.0%) | DPP-4i | 2115 | 15720 | 20.4 (19.6;21.3) | Reference | Reference |  |
|  | GLP-1 RA | 338 | 5836 | 15.8 (14.1;17.5) | 0.70 (0.63;0.79) | 0.93 (0.83;1.05) |  |
| Missing HbA1c information | DPP-4i | 3288 | 15530 | 24.3 (23.6;25.1) | Reference | Reference |  |
|  | GLP-1 RA | 427 | 3582 | 16.6 (15.1;18.0) | 0.63 (0.57;0.70) | 0.92 (0.83;1.02) |  |
|  |  |  |  |  |  |  |  |
| **Achieved HbA1c category** | |  |  |  |  |  |  |
| HbA1c < 48 mmol/mol (< 6.5%) | DPP-4i | 133 | 821 | 21.3 (18.0;24.6) | Reference | Reference | 0.75 |
|  | GLP-1 RA | 41 | 1104 | 8.6 (5.9;11.3) | 0.41 (0.29;0.58) | 0.75 (0.53;1.12) |  |
| HbA1c 48-53 mmol/mol (6.5-7.0%) | DPP-4i | 352 | 2589 | 20.2 (18.3;22.2) | Reference | Reference |  |
|  | GLP-1 RA | 45 | 1177 | 12.9 (9.1;16.6) | 0.57 (0.42;0.77) | 0.80 (0.59;1.10) |  |
| HbA1c 54-58 mmol/mol (7.1-7.5%) | DPP-4i | 502 | 4001 | 19.6 (10.0;21.3) | Reference | Reference |  |
|  | GLP-1 RA | 60 | 1358 | 14.8 (11.2;18.5) | 0.62 (0.47;0.81) | 0.83 (0.64;1.09) |  |
| HbA1c > 58 mmol/mol (>7.5%) | DPP-4i | 1613 | 11719 | 20.7 (19.8;21.7) | Reference | Reference |  |
|  | GLP-1 RA | 278 | 4478 | 16-.0 (14.1;17.9) | 0.72 (0.63;0.82) | 0.95 (0.83;1.08) |  |
| Missing HbA1c information | DPP-4i | 3288 | 15530 | 24.3 (23.6;25.1) | Reference | Reference |  |
|  | GLP-1 RA | 427 | 3582 | 16.6 (15.1;18.0) | 0.63 (0.57;0.70) | 0.92 (0.83;1.02) |  |

**Table S7**: Risk of the composite outcome according to treatment and achieved level of HbA1c among the population with proportion of days covered prior to inclusion was above 80%. The composite outcome included non-fatal myocardial infarction, non-fatal stroke and all-cause death. * The model was adjusted for the following covariates: age, gender, living alone (yes/no), type 2 diabetes duration, pre-treatment HbA1c category (≤ 53 mmol/mol (≤ 7.0%), >53 mmol/mol (> 7.0%), and missing HbA1c), highest achieved educational level, body mass index category (underweight/normal weight, overweight, missing), smoking status (never smoker, never smoker, ex-smoker, occasional or daily smoker, missing), known comorbidities (atrial fibrillation, cancer, chronic obstructive pulmonary disease, hypertension, chronic kidney disease, cardiovascular disease, heart failure), and inclusion year. Abbreviations: n: number, CI: confidence interval, HbA1c: glycated hemoglobin, DPP-4i: dipeptidyl peptidase-4 inhibitor, GLP-1 RA: glucagon-like-peptide-1 receptor agonist.

|  |  | **N events** | **N persons** | **5-year risk (95% CI)** | **Univariate hazard ratio (95% CI)** | **Adjusted hazard ratio (95% CI)*** | **P value for interaction** |
| --- | --- | --- | --- | --- | --- | --- | --- |
| **Mean HbA1c category** |  |  |  |  |  |  | 0.16 |
| HbA1c ≤ 53 mmol/mol (≤7.0%) | DPP-4i | 769 | 5275 | 22.9 (21.3;24.4) | Reference | Reference |  |
|  | GLP-1 RA | 123 | 3264 | 12.8 (10.4;15.1) | 0.47 (0.39;0.57) | 0.76 (0.63;0.92) |  |
| HbA1c >53 mmol/mol (> 7.0%) | DPP-4i | 1571 | 11360 | 21.2 (20.2;22.2) | Reference | Reference |  |
|  | GLP-1 RA | 214 | 3689 | 15.5 (13.3;17.7) | 0.71 (0.61;0.82) | 0.96 (0.83;1.11) |  |
| Missing HbA1c information | DPP-4i | 4753 | 23204 | 24.9 (24.3;25.5) | Reference | Reference |  |
|  | GLP-1 RA | 617 | 6681 | 16.2 (15.0;17.4) | 0.59 (0.54;0.64) | 0.89 (0.82;0.97) |  |
|  |  |  |  |  |  |  |  |
| **Mean HbA1c category** |  |  |  |  |  |  | 0.45 |
| HbA1c < 48 mmol/mol (< 6.5%) | DPP-4i | 299 | 11729 | 24.8 (22.2;27.4) | Reference | Reference |  |
|  | GLP-1 RA | 67 | 1751 | 11.8 (8.9;14.8) | 0.40 (0.31;0.52) | 0.75 (0.57;0.97) |  |
| HbA1c 48-53 mmol/mol (6.5-7.0%) | DPP-4i | 470 | 3546 | 21.8 (20.0;23.7) | Reference | Reference |  |
|  | GLP-1 RA | 56 | 1513 | 14.1 (10.2:17.9) | 0.53 (0.40;0.70) | 0.75 (0.5;1.0) |  |
| HbA1c 54-58 mmol/mol (7.1-7.5%) | DPP-4i | 500 | 4011 | 19.9 (18.3;21.6) | Reference | Reference |  |
|  | GLP-1 RA | 55 | 1164 | 12.8 (9.0;16.5) | 0.69 (0.52;0.91) | 0.93 (0.70;1.23) |  |
| HbA1c > 58 mmol/mol (>7.5%) | DPP-4i | 1071 | 7349 | 21.9 (20.7;23.2) | Reference | Reference |  |
|  | GLP-1 RA | 159 | 2525 | 16.6 (13.9;19.2) | 0.71 (0.60;0.84) | 0.94 (0.80;1.12) |  |
| Missing HbA1c information | DPP-4i | 4753 | 23204 | 24.9 (24.3;25.5) | Reference | Reference |  |
|  | GLP-1 RA | 617 | 6681 | 16.2 (15.0;17.4) | 0.59 (0.54;0.64) | 0.89 (0.82;0.97) |  |

**Table S8**: Risk of the composite outcome according to treatment and cumulative achieved level of HbA1c. The composite outcome included non-fatal myocardial infarction, non-fatal stroke and all-cause death. * The model was adjusted for the following covariates: age, gender, living alone (yes/no), type 2 diabetes duration, pre-treatment HbA1c category (≤ 53 mmol/mol (≤ 7.0%), >53 mmol/mol (> 7.0%), and missing HbA1c), highest achieved educational level, body mass index category (underweight/normal weight, overweight, missing), smoking status (never smoker, never smoker, ex-smoker, occasional or daily smoker, missing), known comorbidities (atrial fibrillation, cancer, chronic obstructive pulmonary disease, hypertension, chronic kidney disease, cardiovascular disease, heart failure), and inclusion year. Abbreviations: n: number, CI: confidence interval, HbA1c: glycated hemoglobin, DPP-4i: dipeptidyl peptidase-4 inhibitor, GLP-1 RA: glucagon-like-peptide-1 receptor agonist.

|  |  | **N events** | **N persons** | **5-year risk (95% CI)** | **Univariate hazard ratio (95% CI)** | **Adjusted hazard ratio (95% CI)*** | **P value for interaction** |
| --- | --- | --- | --- | --- | --- | --- | --- |
| **Achieved HbA1c level** |  |  |  |  |  |  |  |
| HbA1c ≤ 53 mmol/mol (≤7.0%) | DPP4i | 218 | 688 | 31.8 (28.3;35.3) | Reference | Reference | 0.18 |
|  | GLP-1 RA | 81 | 2853 | 9.6 (7.3;11.8) | 0.29 (0.22;0.37) | 0.72 (0.53;0.97) |  |
| HbA1c >53 mmol/mol (> 7.0%) | DPP4i | 566 | 1972 | 28.7 (26.7;30.7) | Reference | Reference |  |
|  | GLP-1 RA | 286 | 6191 | 13.8 (12.1;15.5) | 0.52 (0.45;0.60) | 0.95 (0.76; 1.17) |  |
| Missing HbA1c information | DPP4i | 2286 | 7599 | 30.1 (29.1;31.2) | Reference | Reference |  |
|  | GLP-1 RA | 251 | 2998 | 13.7 (12.1;15.4) | 0.43 (0.37;0.49) | 0.88 (0.73;1.06) |  |

**Table S9:** Risk of the composite outcome according to treatment and achieved level of HbA1c including DPP-4i users as controls prior to 2012 and GLP-1 RA users after 2012. The composite outcome included non-fatal myocardial infarction, non-fatal stroke and all-cause death. * The model was adjusted for the following covariates: age, gender, living alone (yes/no), type 2 diabetes duration, pre-treatment HbA1c category (≤ 53 mmol/mol (≤ 7.0%), >53 mmol/mol (> 7.0%), and missing HbA1c), highest achieved educational level, body mass index category (underweight/normal weight, overweight, missing), smoking status (never smoker, never smoker, ex-smoker, occasional or daily smoker, missing), known comorbidities (atrial fibrillation, cancer, chronic obstructive pulmonary disease, hypertension, chronic kidney disease, cardiovascular disease, heart failure), and inclusion year. Abbreviations: n: number, CI: confidence interval, HbA1c: glycated hemoglobin, DPP-4i: dipeptidyl peptidase-4 inhibitor, GLP-1 RA: glucagon-like-peptide-1 receptor agonist.

|  |  | **N events** | **N persons** | **5-year risk (95% CI)** | **Univariate hazard ratio (95% CI)** | **Adjusted hazard ratio (95% CI)*** | **P value for interaction** |
| --- | --- | --- | --- | --- | --- | --- | --- |
| **Achieved HbA1c level** |  |  |  |  |  |  |  |
| HbA1c ≤ 53 mmol/mol (≤7.0%) | SU-TZD | 878 | 1954 | 45.0 (42.8;47.2) | Reference | Reference | 0.009 |
|  | GLP-1 RA | 81 | 2849 | 9.6 (7.3;11.8) | 0.20 (0.16:0.25) | 0.56 (0.56;0.74) |  |
| HbA1c >53 mmol/mol (> 7.0%) | SU-TZD | 4308 | 8812 | 49.0 (47.9;50.0) | Reference | Reference |  |
|  | GLP-1 RA | 287 | 6214 | 13.6 (11.9;15.3) | 0.28 (0.25;0.32) | 0.79 (0.65;0.97) |  |
| Missing HbA1c information | SU-TZD | 49 612 | 78847 | 63.0 (62.7;63.3) | Reference | Reference |  |
|  | GLP-1 RA | 259 | 3022 | 14.0 (12.4;15.6) | 0.17 (0.15;0.19) | 0.65 (0.54;0.77) |  |

**Table S10:** Risk of the composite outcome according to treatment and achieved level of HbA1c including sulfonylurea and thiazolidinedione users as reference group. The composite outcome included non-fatal myocardial infarction, non-fatal stroke and all-cause death. * The model was adjusted for the following covariates: age, gender, living alone (yes/no), type 2 diabetes duration, pre-treatment HbA1c category (≤ 53 mmol/mol (≤ 7.0%), >53 mmol/mol (> 7.0%), and missing HbA1c), highest achieved educational level, body mass index category (underweight/normal weight, overweight, missing), smoking status (never smoker, never smoker, ex-smoker, occasional or daily smoker, missing), known comorbidities (atrial fibrillation, cancer, chronic obstructive pulmonary disease, hypertension, chronic kidney disease, cardiovascular disease, heart failure), and inclusion year. Abbreviations: n: number, CI: confidence interval, HbA1c: glycated hemoglobin, SU: sulfonylurea, TZD: thiazolidinedione, GLP-1 RA: glucagon-like-peptide-1 receptor agonist.


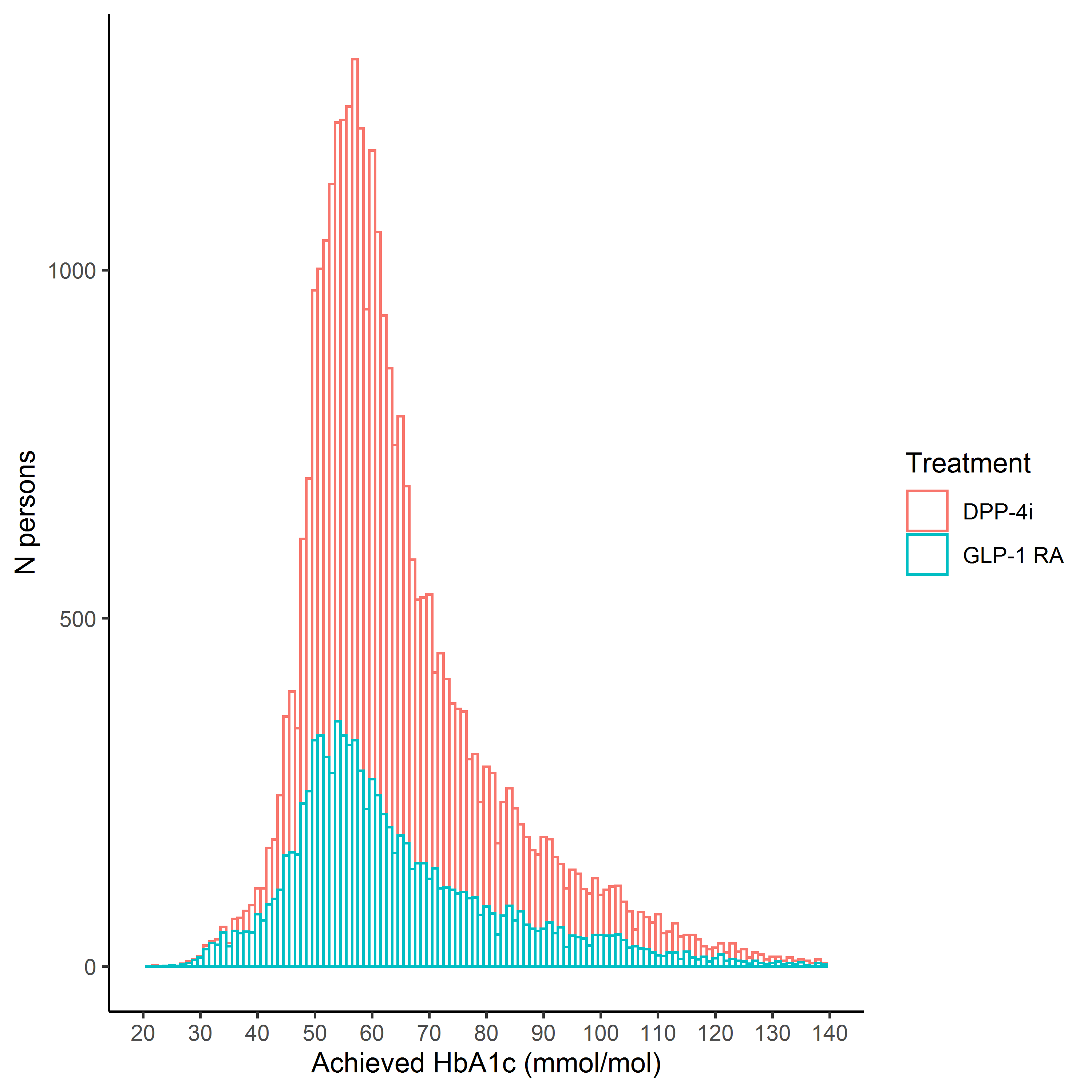


**Figure S1:** The frequency distribution of the achieved HbA1c values (mmol/mol) among those with a HbA1c measurement within six months following initiation of treatment with DPP-4i or GLP-1 RA.


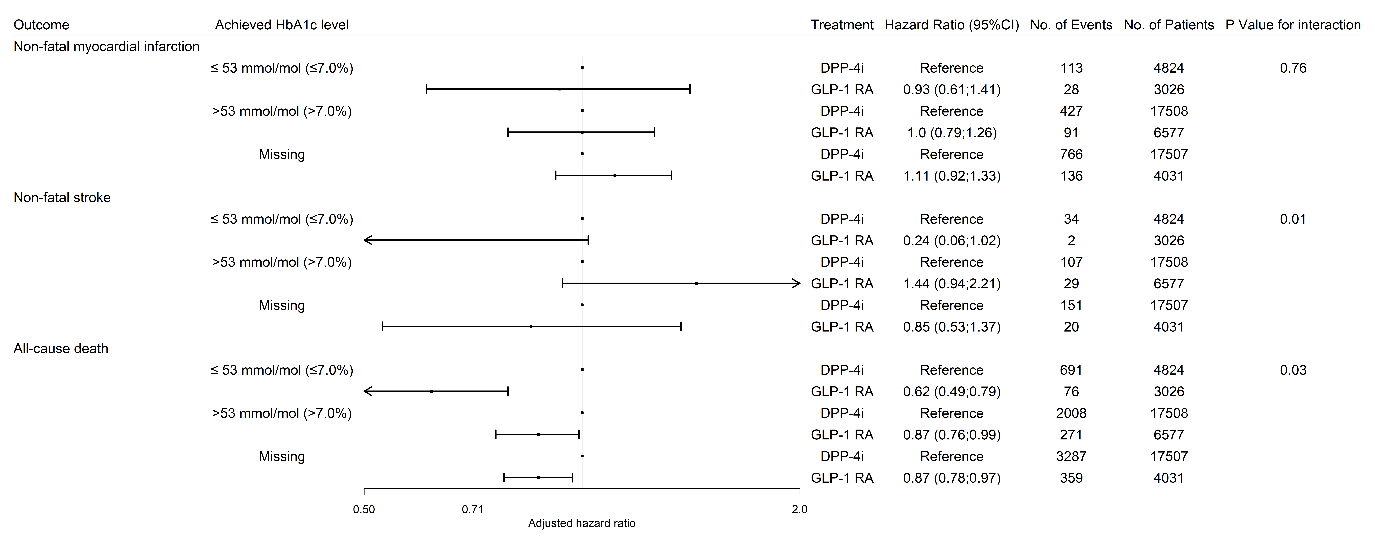


**Figure S2**: Risk of the individual components of the composite outcome, according to treatment and achieved HbA1c level. The composite outcome included non-fatal myocardial infarction, non-fatal stroke and all-cause death. * The model was adjusted for the following covariates: age, gender, living alone (yes/no), type 2 diabetes duration, pre-treatment HbA1c category (≤53 mmol/mol (≤ 7.0%), >53 mmol/mol (> 7.0%), and missing HbA1c), highest achieved educational level, body mass index category (underweight/normal weight, overweight, missing), smoking status (never smoker, never smoker, ex-smoker, occasional or daily smoker, missing), known comorbidities (atrial fibrillation, cancer, chronic obstructive pulmonary disease, hypertension, chronic kidney disease, cardiovascular disease, heart failure), and inclusion year. Abbreviations: HbA1c: glycated haemoglobin, N: number, CI: confidence interval.

**
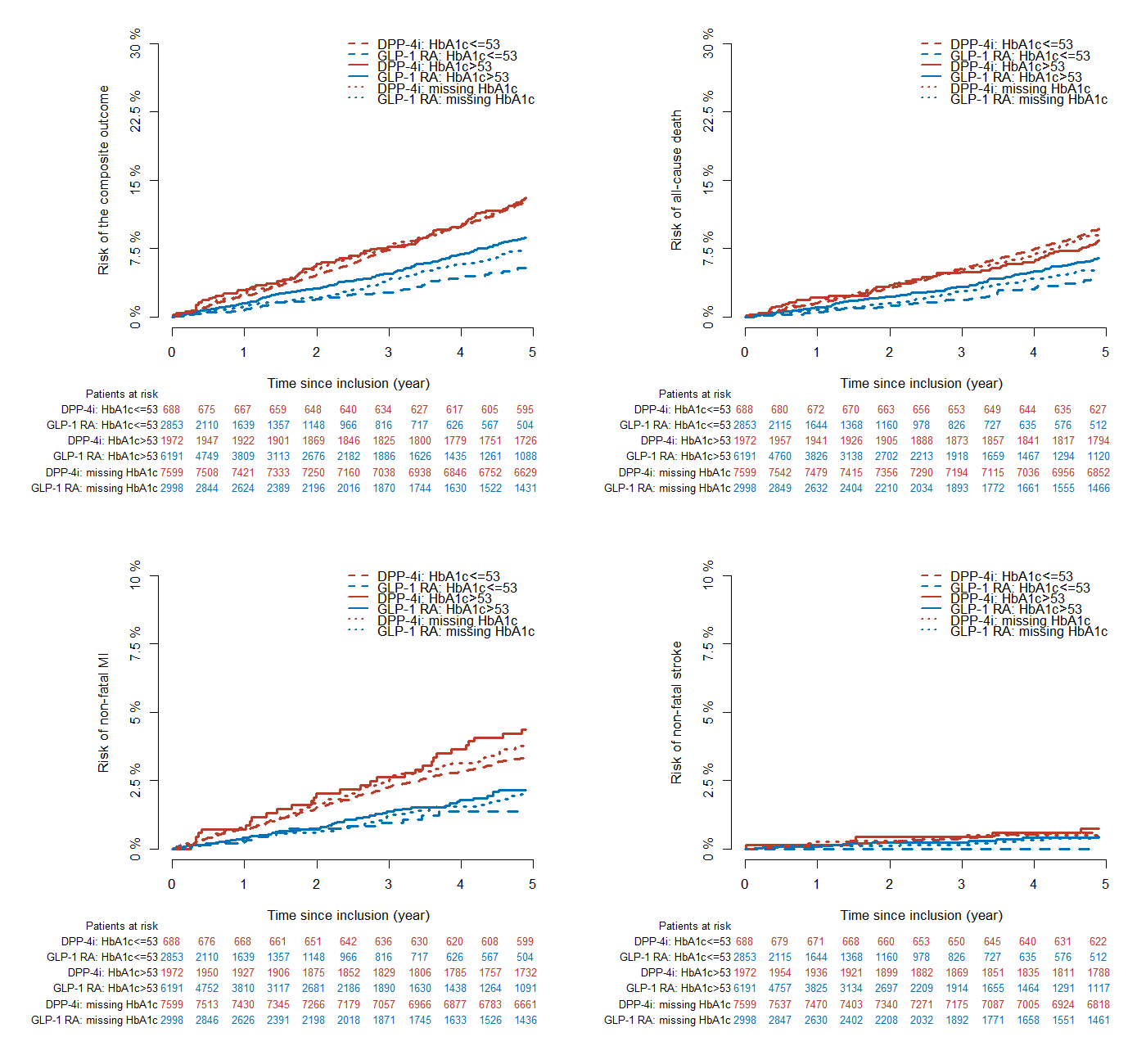
**

**Figure S3:** Risk of the composite outcome and components of the composite outcome (non-fatal myocardial infarction, non-fatal stroke, and all-cause death) according to treatment and achieved HbA1c level (mmol/mol) among DPP-4i users prior to 2012 and GLP-1 RA users after 2012. Abbreviations: MI: myocardial infarction: HbA1c: glycated hemoglobin, DPP-4i: dipeptidyl peptidase-4 inhibitor, GLP-1 RA: glucagon-like-peptide-1 receptor agonists.

**
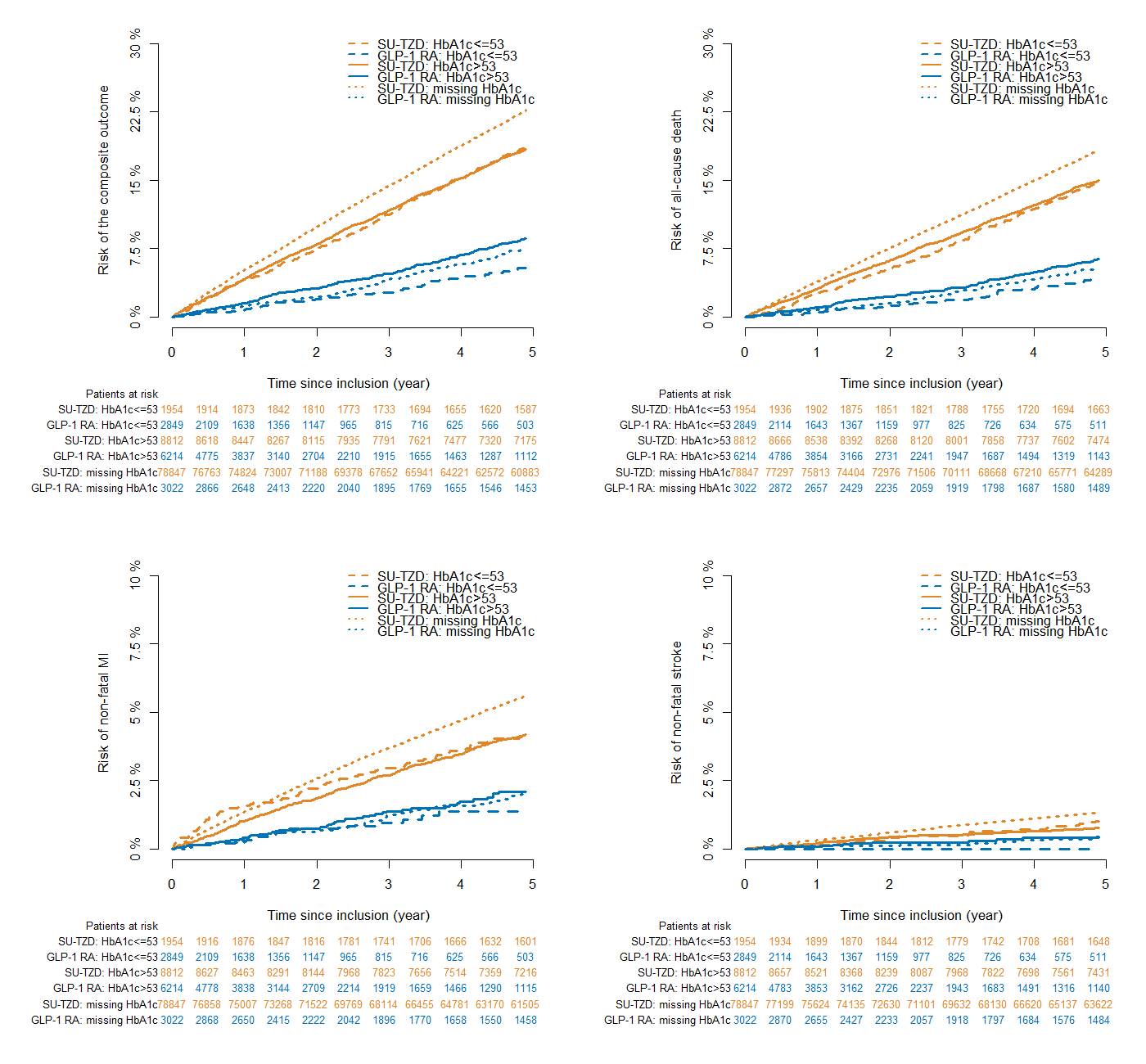
**

**Figure S4:** Risk of the composite outcome and components of the composite outcome (non-fatal myocardial infarction, non-fatal stroke, and all-cause death) according to treatment and achieved HbA1c level (mmol/mol) among sulfonylurea and thiazolidinedione users prior to 2012 and GLP-1 RA users after 2012. Abbreviations: MI: myocardial infarction: HbA1c: glycated hemoglobin, SU: sulfonylurea, TZD: thiazolidinedione, GLP-1 RA: glucagon-like-peptide-1 receptor agonists.
